# When are novel methods for analyzing complex chemical mixtures in epidemiology beneficial?

**DOI:** 10.1097/EE9.0000000000000456

**Published:** 2026-01-14

**Authors:** Nate Wiecha, Emily Griffith, Brian J. Reich, Jane A. Hoppin

**Affiliations:** a Department of Statistics, North Carolina State University, Raleigh, North Carolina; b Department of Biological Sciences, North Carolina State University, Raleigh, North Carolina

**Keywords:** Chemical mixtures, Epidemiology, Simulation, Hypothesis testing

## Abstract

Estimating the health impacts of exposure to a mixture of chemicals poses many statistical challenges: multiple correlated exposure variables, moderate to high dimensionality, and possible nonlinear and interactive health effects of mixture components. Reviews of chemical mixture methods aim to help researchers select a statistical method suited to their goals and data, but examinations of empirical performance have emphasized novel methods purpose-built for analyzing complex chemical mixtures, or other more advanced methods, over more general methods that are widely used in many application domains. We conducted a broad experimental comparison, across simulated scenarios, of both more general methods (such as generalized linear models) and novel methods (such as Bayesian Kernel Machine Regression) designed to study chemical mixtures. We assessed methods based on their ability to control the Type I error rate, maximize power, provide interpretable results, and make accurate predictions. We find that when there is moderate correlation between mixture components and the exposure-response function does not have complicated interactions, or when mixture components have opposite effects, general methods are preferred over novel ones. With highly interactive exposure-response functions or highly correlated exposures, novel methods provide important benefits. We provide a comprehensive summary of when different methods are most suitable.

**What this study adds**

Our review and simulation study comparing chemical mixture methods differs from others because we consider general methods such as generalized linear models alongside specialized methods; we consider simpler data-generating scenarios in our simulations alongside more complex ones; and we center Type I error rate control in our recommendations due to its importance to statistical hypothesis testing. Previous simulation studies in this domain have generally considered more complex methods applied to more complex data-generating scenarios, and have not as strongly emphasized the importance of Type I error rate control. We are, therefore, to our knowledge, the first review of chemical mixture methods to offer clear empirical guidance on choosing a method to researchers whose primary goal is statistical hypothesis testing and whose data may not have arisen from a highly complex data-generating process.

## Introduction

Observational studies analyzing the association between a mixture of multiple exposures and a health outcome face unique statistical challenges. Examples are Lebeaux et al,^1^ which studied the association between maternal serum concentrations of four per- and polyfluoroalkyl substances (PFAS) and thyroid hormone levels in maternal and cord serum, and Bobb et al,^2^ which used as a motivating example an analysis of the association between concentration of arsenic, manganese, and lead in umbilical cord blood and a psychomotor development score in early childhood. Including multiple relevant exposures in a model avoids left-out variable bias, but the levels of different exposures may be correlated due to shared sources, making it difficult to distinguish effects. The number of exposures analyzed can be moderate to large, for example, 11 exposures in Carrico et al,^3^ to over 100 in Maitre et al.^4^ Effects of exposures on health outcomes can be nonlinear and interactive, making model specification challenging.

A number of novel methods, designed specifically for analyzing chemical mixtures by dealing with one or more of these difficulties, have been proposed (see Section “Statistical methods for chemical mixtures”). For our review, we define methods as “novel” chemical mixture methods if they were designed specifically for this application, while we consider “general” methods to be those in wide use in a variety of applications. Several reviews have summarized different methods, models, and considerations for researchers.^5,6^ Several include simulation studies of properties of some methods, primarily novel chemical mixture methods, or other advanced methods which are not in wide use in applications.^7–9^ To our knowledge, recommendations so far have therefore either not included simulation studies or included simulation studies but did not include situations where simpler or more general methods may perform better than more complicated or novel methods. Simulations so far have also not treated Type I error rate control as a critical consideration.^7–9^ The resulting incomplete picture of mixture methods’ empirical performance may bias researchers’ choices of method.

Our study assesses the performance of novel chemical mixture methods and more general methods, so that researchers may understand what method best meets their needs. We compare methods’ performance on Type I error rate control, power, and predictive accuracy, in an experimental manner across a range of simulated scenarios meant to model realistic situations. We also examine qualitative aspects of model choice, such as additional assumptions and interpretability. While we focus on exposure to chemical mixtures such as PFAS, our results are applicable to any study with similar statistical difficulties, such as studies involving multiple air pollutants or other independent variables that may be correlated.

Section “Statistical setting” outlines the statistical setting; Section “Statistical methods for chemical mixtures” describes several chemical mixture methods; Section “Simulation study” presents the simulation study design; Section “Results” presents results of the simulation; and Section “Discussion” presents our discussion and recommendations.

### Statistical setting

The generic setting is trying to estimate parameters or characteristics of an exposure-response function *h*, where, letting $\mu_{i}\equiv E(y_{i}|a_{i},x_{i})$:


$$\begin{array}{c} \eta_{i}=g\left(\mu_{i}\right)=h\left(a_{i1},\;\ldots ,\;a_{ip}\right)+x_{i}^{T}\beta_{X}, \end{array}$$ (1)

where $a_{ij}$ denotes the value of exposure *j* for observation *i*, $x_{i}$ is the covariate vector for observation *i*, and *g* is a monotonic, differentiable link function. With an assumed distribution for $y_{i}|a_{i},x_{i}$, different choices of g are used to model different types of data. Common cases are:


$$y_{i}\sim N\left(\mu_{i},\sigma^{2}\right),\;\;\;\;\eta_{i}=g\left(\mu_{i}\right)=\;\mu_{i},$$ (2)

$$y_{i}\sim Ber\left(\mu_{i}\right),\;\;\;\eta_{i}=g\left(\mu_{i}\right)=\ln\left(\frac{\mu_{i}}{1-\mu_{i}}\right),\;$$ (3)

$$y_{i}\sim Pois\left(\mu_{i}\right),\;\;\;\eta_{i}=g\left(\mu_{i}\right)=\ln\left(\mu_{i}\right),\;$$ (4)

where (2) models Gaussian-distributed continuous outcome data using the identity link function, (3) models Bernoulli-distributed binary outcome data using the logistic link function, and (4) models Poisson-distributed count outcome data using the log link function. For generality, we discuss models for the expected outcome on the scale of the link function, *η*_*i*_ = *g*(*µ*_*i*_). All methods in Section “Statistical methods for chemical mixtures” can model at least Gaussian and binary data.

We assume the exposure variables have continuous values, such as measured concentrations of chemicals in blood serum. For notational simplicity, we do not include covariates in the models.

## Statistical methods for chemical mixtures

Methods used for analyzing chemical mixtures may seek to accomplish any of three goals: (1) identifying elements of the mixture that are associated with the response, (2) identifying whether the mixture as a whole is associated with the response, and (3) make predictions on new (out-of-sample) data.

### General methods

#### Generalized linear models

Generalized linear models (GLMs) assume that on the scale of the link function *g*, the expected value of the response is linearly related to the predictors. A GLM takes the form:


$$\eta_{i}=\;\beta_{0}+\;a_{i}^{T}\beta .$$

Nonlinearities and interactions must be modeled explicitly by the user, by including higher-order terms or transformations of the exposure variables as predictors in the model. Correlation between exposures can be addressed using an *F*-test or *χ*^2^-test for joint association between any of the exposures and the response, testing *H*_0_: *β*_1_ = *β*_2_ = *…* = *β*_*p*_ = 0. When covariates are included in the model, a partial *F*-test can be used to only test for the association between the exposures and response, excluding covariate effects from the null hypothesis. Interpretation of GLMs is easy: *β*_*j*_ represents the change in *η*_*i*_ = *g*(*µ*_*i*_) per unit change in *a*_*ij*_. Functions to fit GLMs are included in R’s base functionality.

#### Principal components regression

Principal components regression (PCR) reduces the dimension of the exposure vector **a**_*i*_ using principal components analysis before regressing *y*_*i*_ onto the new, derived explanatory variables.^10^ In some cases, the new variables can represent underlying features of the data. In these cases, in high-dimensional problems, and where the assumption of linearity is reasonable, PCR may be useful. It also may be useful when the exposures are highly correlated because the derived variables are orthogonal. The original exposure variables are scaled, and then principal components analysis obtains *M* ≤ *p* principal components (PCs) from the scaled exposures. The first PC explains the most variance in the scaled exposures, followed by the rest of the PCs in sequence. Often, a small number of PCs can approximate the scaled exposures. Then the model is


$$\begin{array}{c} \eta_{i}=\beta_{0}+z_{i}^{T}\theta , \end{array}$$ (5)

where the *M*-vector of PC values for observation *i* is **z**_*i*_. Testing *H*_0_: *θ*_1_ = *…* = *θ*_*M*_ = 0 tests for any association between the components of the mixture and the response. When covariates are included, the dimension reduction step should not be applied to the covariates, and a partial *F*-test can be used to only test for association between the principal components and the response, excluding covariate effects from the null hypothesis. This test does not account for uncertainty in determining the PCs or which PCs to include in the regression model.

The PCs are weighted sums of the scaled exposure variables. If there are underlying features of the exposure data, such as a shared source of multiple exposures, PCs may correspond to these features, although they must be interpreted by the user based on the exposure weights. It is not possible to conduct hypothesis tests for the association of the individual chemicals with the outcome variable.

An exposure may be only lightly weighted in the selected PCs, but contribute substantially to the response, causing PCR to miss an important component of the exposure-response relationship, a risk not quantified by the fitted model. PCR is easily implemented using functions included in base R.

#### LASSO/Elastic net regression

Least Absolute Shrinkage and Selection Operator (LASSO) regression and elastic net regression are linear models that perform variable selection rather than hypothesis testing, suiting them to high-dimensional settings when statistical inference is less important than prediction.^10^ The model is again $\eta_{i}=\;\beta_{0}+a_{i}^{T}\beta .$ The LASSO estimator for the identity link and Gaussian likelihood is


$$\begin{array}{c} {\hat{\beta }}_{LASSO}=\mathrm{\backslash arg}\underset{\beta }{\min}\;\left\{\underset{i=1}{\overset{n}{\sum }}\;{\left(Y_{i}-\beta_{0}-a_{i}^{T}\beta \right)}^{2}+\lambda \underset{j=1}{\overset{p}{\sum }}\;\left|\beta_{j}\right|\right\}, \end{array}$$ (6)

and the elastic net estimator is


$$\begin{array}{c} {\hat{\beta }}_{EN}=\mathrm{\backslash arg}\underset{\beta }{\min}\;\left\{\underset{i=1}{\overset{n}{\sum }}\;{\left(Y_{i}-\beta_{0}-a_{i}^{T}\beta \right)}^{2}+\lambda \left(\alpha \underset{j=1}{\overset{p}{\sum }}\;\left|\beta_{j}\right|+\left(1-\alpha \right)\underset{j=1}{\overset{p}{\sum }}\;\beta_{j}^{2}\right)\right\} \end{array}$$ (7)

with *λ >* 0*, α* ∈ [0,1]. Typically, cross-validation is used to select *λ* and *α*.^10^ The penalty term $\underset{j=1}{\overset{p}{\sum }}\;\left|\beta_{j}\right|$ in (6) and (7) results in the *β*_*j*_ being constrained to a *p*-dimensional region with corners along the coordinate axes; as a result, the minima in (6) and (7) may be attained at these corners, setting some of the *β*_*j*_ to be zero and performing variable selection.^10^

Elastic net regression has sometimes been preferred in the analysis of exposure mixtures because LASSO has a tendency to arbitrarily select from among a group of correlated exposures, while elastic net tends to either exclude or include correlated exposure variables together.^3^ However, groupings under elastic net come from patterns of exposure and not associations with the response. These methods’ selections can be unstable.^11^ Coefficient estimates obtained after selecting variables are biased and typically do not reflect uncertainty in model selection.^10,12^

An R package used to fit elastic net models, including LASSO, including for discrete outcome data, is glmnet.^13^

#### Generalized additive models

Generalized additive models (GAMs) flexibly model the exposure-response relationship *h* with simplifying assumptions. A GAM that is additive in the exposures is


$$\eta_{i}=\beta_{0}+\underset{j=1}{\overset{p}{\sum }}\;f_{j}\left(a_{ij}\right),$$

where *β*_0_ is the intercept, and *f*_*j*_*, j* = 1*,...,p* is an unknown smooth function of the *j*th exposure. Typically, the unknown functions are approximated using linear combinations of *k* spline basis functions, so that the model is


$$f_{j}\left(a_{ij}\right)\approx \underset{r=1}{\overset{k}{\sum }}\;B_{jr}\left(a_{ij}\right)\beta_{jr},$$

with $B_{jr}\left(\cdot \right)$, *r* = 1*,...,k* denoting the B-spline basis functions used to approximate each function *f*_*j*_ as in Wei et al.^14^ Typically, a smoothing penalty is applied when estimating the *β*_*jr*_ to prefer smoother estimates of the *f*_*j*_. GAMs can be interpreted by plotting the fitted functions. Including interactions is possible but often impractical except with low *p*.

Frequentist hypothesis tests for the statistical significance of smooth terms are available in software such as the R package mgcv.^15^ Per Wood, frequentist hypothesis tests may perform poorly when exposures are correlated, and better hypothesis test results are obtained by selecting the smoothing penalty using restricted maximum likelihood.^16^ Alternatively, penalties similar to those in LASSO (Section “LASSO/Elastic net regression”) can be used to perform variable selection. The implementations of GAMs in mgcv are highly optimized and computationally efficient.^15^

### Novel methods for complex chemical mixtures

#### Bayesian kernel machine regression

Bayesian kernel machine regression (BKMR) is a fully Bayesian implementation of Gaussian Process (GP) regression of the response onto the exposures.^2^ The flexible GP model allows BKMR to estimate the exposure-response function even in the presence of nonlinear and interaction effects. BKMR uses a variable-selection before determine which components are associated with the response and estimates the model using Markov Chain Monte Carlo. There are two approaches to variable selection available. The componentwise method performs variable selection on each individual mixture component. The hierarchical variable selection method performs variable selection on prespecified groups of mixture components, such as groups of highly correlated components.

The Bayesian model for *η*_*i*_ with component-wise variable selection is:


$$\begin{array}{c} \eta_{i}=h_{i}=h\left(a_{i1},\;\ldots ,\;a_{ip}\right), \end{array}$$ (8)

$$h\equiv {\left(h_{1},\ldots ,\;h_{n}\right)}^{T}|K\sim N\left(0,\;\tau K\right)$$

$$K_{\ell m}\equiv K\left(a_{\ell },\;a_{m};r\right)\equiv \exp\left\{-\underset{j=1}{\overset{p}{\sum }}\;r_{j}{\left(a_{\ell j}-a_{mj}\right)}^{2}\right\}.$$

The unknown exposure-response function *h* is given a GP prior with kernel matrix **K**, defined through the kernel function K(·,·)  , which determines the correlation between values of *h* corresponding to different values of the exposures. BKMR scales the squared difference between *a*_*ℓj*_*, a*_*mj*_ (the *ℓ*th and *m*th observations of the *j*th exposure) by *r*_*j*_ for each *ℓ* ≠ *m*, and variable selection is performed by giving *r*_*j*_ a spike-and-slab prior with a spike at 0, for *j* = 1*,...,p*. When *r*_*j*_ = 0, the *j*th exposure variable is deselected from the model, as it has no effect on **K** and therefore on the estimate of *h*. The posterior inclusion probability (PIP) for exposure *j* is the posterior probability that *r*_*j*_ is nonzero.^2^ The hierarchical variable selection model yields a PIP for each group of variables, and then ranks the variables within each group by importance.

Much of BKMR’s interpretation uses graphical summaries of the posterior distribution of *h*. Statistical inference is done most directly using the PIPs of the *p* exposure components. Contrasts can also be estimated and tested using the posterior distribution of *h*, to test hypotheses related to main, joint, or interaction effects of the different chemicals.^17^ Inference based on findings from graphical analysis of the posterior involves multiple comparisons, possibly many comparisons, and PIPs can be sensitive to prior specification. BKMR is implemented in the R package bkmr.

#### Weighted quantile sum regression

Weighted quantile sum (WQS) regression removes the effects of multicollinearity by considering the mixture as a whole.^3^ WQS regression forms a weighted sum, or index, of the exposure variables, and regresses the outcome onto the index. WQS regression uses the quantized versions of the exposure variables as inputs to the index, termed the “weighted quantile sum.” Let *q*_*ij*_ denote the values of the quantized exposure variables. For example, *q*_*ij*_ would be the quantized value of *a*_*ij*_, so that if sample quartiles are used, *q*_*ij*_ could take values 1, 2, 3, or 4. The WQS model is


$$\begin{array}{c} \eta_{i}=\beta_{0}+\beta_{1}\left(\underset{j=1}{\overset{p}{\sum }}\;w_{j}q_{ij}\right), \end{array}$$ (9)

where *w*_*j*_ is the weight assigned to exposure variable *j*. Inference on the association of the mixture with the response is performed by testing *H*_0_: *β*_1_ = 0. The sample is by default split into training and validation sets, with the training set used to estimate the weights, and the validation set used to test *H*_0_. WQS regression assumes that all exposures have either nondecreasing or nonincreasing associations with the outcome, an assumption sometimes termed “directional homogeneity.”^18^ WQS regression is implemented in the R package gWQS.

#### Quantile g-computation

Similar to WQS regression, quantile g-computation (QGC) assesses the association of the entire mixture with the response.^18^ The model is


$$\begin{array}{c} \eta_{i}=\;\beta_{0}+\;\underset{j=1}{\overset{p}{\sum }}\;\beta_{j}q_{ij}, \end{array}$$ (10)

where the notation is as above. Inference is performed on the quantity $\psi =\;\sum_{j=1}^{\;p}\beta_{j},$ interpreted as the expected increase in the outcome when increasing the level of every mixture component by one quantile. Model (10) can be extended to include nonlinear or product terms of the exposures. Weights for each exposure are defined by separately normalizing the positive and negative regression coefficient estimates to sum to 1, which can be misleading. QGC regression is implemented in the R package qgcomp.

## Simulation study

In our simulation study, different methods are evaluated on: (1) their ability to control Type I error probability, (2) their power to detect association of an individual component with the response, (3) their power to detect association of the mixture with the response, and (4) predictive accuracy measured by mean squared error (MSE) on new data. We evaluated four general methods and three novel mixture methods, including several variations, on these criteria. Details of the methods’ implementation are in the Supplementary Materials Section S1; https://links.lww.com/EE/A398. Abbreviations used in results tables and figures are listed in Table 1.

**Table 1.** Abbreviations for methods used in the simulation study

Abbreviation	Method and notes
GLM	Generalized linear model, using individual hypothesis tests to test the significance of individual components or *F*-test to test the significance of the mixture. Used with the identity link function and Gaussian outcome distribution, that is, ordinary least-squares regression
ENET	Elastic net, using variable selection to select individual components
PCR	Principal components regression, using an *F*-test to test the significance of the mixture, selecting enough components to explain 75% of the overall variance in exposures
GAM (HT)	GAM using frequentist hypothesis testing to test the significance of individual components
GAM (VS)	GAM using variable selection to select individual components
GAM (HT, Bonf.)	GAM using Bonferroni’s correction of individual component hypothesis tests to test the significance of the mixture
BKMR (Contrast)	BKMR testing for the significance of the mixture using an estimated contrast between levels of overall exposure
BKMR (Hier, 0.95)	BKMR testing for the significance of the mixture using hierarchical variable selection, and a PIP at least 0.95 indicating significance
BKMR (0.50)	BKMR testing the significance of individual components, and a PIP at least 0.50 indicating significance.
BKMR (0.95)	BKMR testing the significance of individual components, and a PIP at least 0.95 indicating significance
QGC	Quantile g-computation, testing the significance of the mixture
WQS	Weighted quantile sum regression, testing the significance of the mixture, assuming positive associations with all individual components

General methods are listed in the top half of the table, followed by novel chemical mixture methods.

### Data-generating process

The sample size *n* was either 100 or 400, and the number of exposure variables *p* was either 5, 10, or 20. The exposures were generated randomly such that each had an Exp(1) marginal distribution, and the correlation *ρ* between each pair of exposures was 0, 0.5, or 0.9.

The response variable *y*_*i*_ was generated as:


$$y_{i}=h\left(a_{i};\;\beta \right)+\epsilon_{i},$$

$$\epsilon_{i}\sim N\left(0,\;2^{2}\right),\;i.i.d.$$

In the main simulations, the true exposure-response function *h* was linear, nonlinear, linear-interactive, or sinusoidal (which is both nonlinear and interactive):


$$h_{lin}\left(a;\;\beta \right)=\beta a_{1}+\beta a_{2},$$

$$h_{nonlin}\left(a;\;\beta \right)=\beta a_{1}^{2}+\beta a_{2}^{2},$$

$$h_{int}\left(a;\;\beta \right)=\beta a_{1}a_{2},$$

$$h_{sine}\left(a;\;\beta \right)=\beta \sin\left(\frac{\sqrt{a_{1}^{2}+a_{2}^{2}}}{2}\right).$$

In these main scenarios, only two exposure variables were related to the response regardless of the value of *p*. In an additional scenario we used $h_{opp}\left(a_{1},a_{2};\beta \right)=\beta a_{1}+a_{2}\;$ and varied *β* from −1 to 1, so that components’ effects could cancel out. In a final “dense” scenario, all exposures affected the response, with $h_{dense}\left(a_{1},\ldots ,\;a_{p};\beta \right)=a^{T}\beta$ where $\beta ={\left(\beta ,...,\;\beta \right)}^{T}$. We tested two hypotheses to evaluate different methods, *H*_0_: *a*_1_ unassociated with *y*, and *H*_0_: no association between the mixture and response.

Values of *β* ranged from 0 to an endpoint informative for each scenario. Prediction mean-squared error was assessed using 100 new points drawn from the same distribution as the samples used for model fitting, except without the noise term *ϵ*_*i*_ added. For *n* = 100, we used 400 Monte Carlo (MC) iterations for each value of *β* in each scenario, and for *n* = 400, we used 200 MC iterations. In each scenario, the estimated Type I error rate was the proportion of MC iterations in which the method wrongly determined the component or mixture was associated with the response when *β* = 0. For frequentist hypothesis tests, if *P*-values were less than the nominal value α=0.05, the null hypothesis was rejected; decision rules for other methods are described in Table 1. Nominal Type I error rate for methods based on frequentist hypothesis testing was therefore α=0.05. The estimated power for each value of *β*≠ 0 was the proportion of MC iterations in which the method correctly determined that the component or mixture is associated with the response.

## Results

Results showed generally that when the correlation between exposures, number of exposures, and/or complexity of the exposure-response function was high, then the novel methods had higher power than general methods. In scenarios where correlation was moderate or low, there were fewer exposures, and the exposure-response function was not too complicated, the general methods were more powerful. Method recommendations based on different scenarios or priorities for the data analysis are presented in Table 2. Detailed summary tables of the strengths and weaknesses we found of each method are in Tables 3 and 4.

**Table 2.** Preferred methods for various analysis priorities that may arise in the analysis of chemical mixtures’ associations with health outcomes

Analysis priority	Preferred method(s)
High correlation between exposures and/or many exposures	QGC, WQS, PCR
Moderate correlation between exposures and/or a moderate number of exposures	GLMs, GAMs
Nonlinear exposure-response function	GAMs
Interactive exposure-response function	BKMR
Interpretable results	GLMs, QGC
Opposite effects of exposures	GLMs, GAMs
Low sample size	All but BKMR

Analysis priorities may be dealing with particular challenges posed by the data, or more qualitative priorities such as the need for interpretable results.

**Table 3.** Detailed summary of different mixture methods’ empirical performance

Method	Nonlinearity, interactivity	Multiple correlated exposures	Identify individual components	Overall performance
Generalized linear model (GLM)	Poor: user-specified model	OK: joint tests; good with low-moderate *p* and *ρ*	Good if *p* not too high	Good if the model approximately correct
Generalized additive model with hypothesis tests (GAM)	Good for nonlinearity; interactivity impractical except for low *p*	Poor: loss of power with correlated predictors	Good if *p* not too high	Good
Elastic net (ENET) (including LASSO)	Poor: user-specified model	OK: good for sparse effects, but ENET tends to (de)select correlated groups, LASSO will select within groups arbitrarily; does not control Type I error	Excellent: Unimportant components deselected, but no uncertainty quantification	Poor Type I error control. If that is unimportant, good performance
Weighted quantile sum (WQS)	OK: quantized variables may implicitly model nonlinearity; user-specified model	Excellent if only interested in the whole mixture; requires directional homogeneity	OK: Gives weights on each component, but no statistical testing	Good if assumptions met, but loses power by splitting sample
Quantile g-computation (QGC)	OK: quantized variables may implicitly model nonlinearity; user-specified model	Excellent if only interested in whole mixture; suffers without directional homogeneity	Good if using individual regression coefficients. Outputted weights may be misleading	Good if assumptions met; does not split the sample
Bayesian kernel machine regression (BKMR)	Excellent: if enough signal/data	OK: use hierarchical model PIPs with caution	OK: PIPs are sensitive to prior, appropriate cutoff for significance is unclear, should be used with care	Good with interaction effects. PIP cutoff of 0.50 oversensitive. Default prior for hierarchical selection is poorly calibrated
Principal components regression (PCR)	Poor: user-specified model	Good, as it combines exposures; however, the combination ignores the outcome, so can ignore important components	Poor: the principal components are difficult to interpret except in limited circumstances	Good for whole-mixture tests, although will fail to identify components not included in the PCs used

*p* denotes the number of exposure variables (mixture components), and *ρ* denotes their pairwise correlations.

LASSO indicates Least Absolute Shrinkage and Selection Operator.

**Table 4.** Detailed summary of different mixture methods’ interpretability, limitations, software, and recommended use

Method	Interpretability	Limitations	R implementation	When to use
Generalized linear model (GLM)	Excellent: coefficients have clear meaning if assumptions satisfied	Strict parametric assumptions	Base R	Low-to-moderate *p*, so specifying a model is reasonable
Generalized additive models with hypothesis tests (GAM)	Good: can easily examine plots of fitted functions	No practical joint test, limited ability to model interaction	mgcv	Nonlinearity present, but whole-mixture test unimportant
Elastic net (ENET) (including LASSO)	OK: coefficients have meaning, but are biased and lack uncertainty quantification	Poor Type I error control; no uncertainty quantification; user-specified model	glmnet; caret	Many predictors, and Type I error control unimportant
Weighted quantile sum (WQS)	OK: Index effect difficult to interpret, but weights have clear meaning	User-specified model; directional homogeneity requirement; limited interpretability; sample splitting	gWQS	Moderate-to-many correlated predictors and individual components unimportant, directional homogeneity
Quantile g-computation (QGC)	OK: Index effect interpretable, but weights misleading; users can interpret initial regression fit	Suffers without directional homogeneity; user-specified model	qgcomp	Moderate-to-many correlated predictors and individual components unimportant, directional homogeneity
Bayesian kernel machine regression (BKMR)	Good qualitatively: detailed graphical summaries of posterior. OK quantitatively: can estimate contrasts that may be crude summaries	Nonparametric: prefers larger *n*; PIPs sensitive to prior tuning and lack guidance on cutoff; examining posterior involves multiple comparisons	bkmr	Lot of data or strong signal, or improved (cross-validated) predictions over other methods
Principal components regression (PCR)	OK: In some situations, PCs may correspond to features of exposure data. Otherwise, poor	Dimension reduction ignores relationship with outcome; poor interpretability	Functions required are in base R	Many components; individual components unimportant or a PC is meaningful; can risk missing important components

*p* denotes the number of exposure variables (mixture components), and *ρ* denotes their pairwise correlations.

LASSO indicates Least Absolute Shrinkage and Selection Operator.

A selection of simulation study results is included in Figures 1–5. Remaining results are in the Supplemental Materials Figures S1–S28; https://links.lww.com/EE/A398 and Tables S1–S16; https://links.lww.com/EE/A398. The power curves display the probability of rejecting the null hypothesis on the *y*-axis, for the value of *β* on the *x*-axis. When *β* = 0, the value of the curve is the Type I error probability (targeting 0.05), since when *β* = 0, there is no association between the exposures and response. Therefore, the power curve for a method that outperforms competitors has a *y*-intercept at a maximum 0.05, and then the rest of its curve above those of other methods. The prediction error curves display the prediction MSE, using predictions on new data not included in the original sample, for the value of *β* on the *x*-axis.

**Figure 1.**
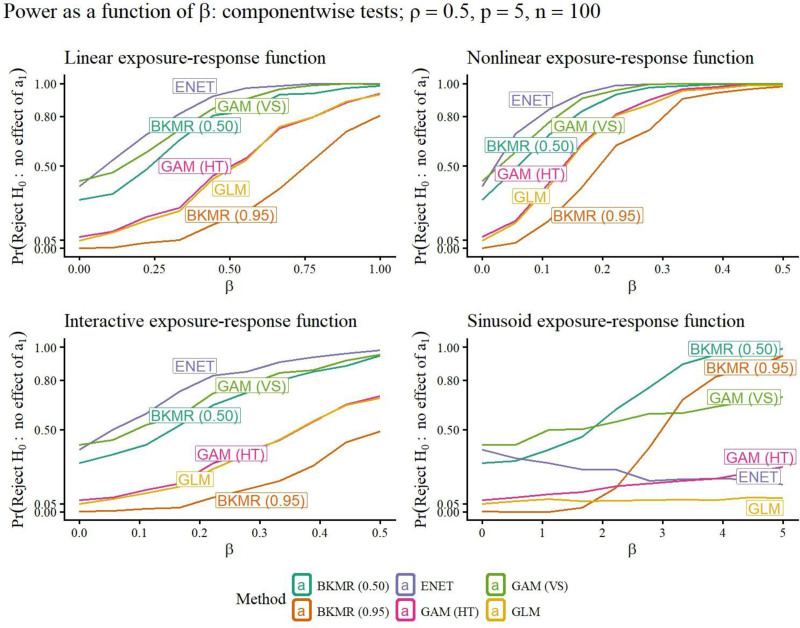
For each method (curve), the y-value is the probability of rejecting *H*_0_: first mixture component *a*_1_ not associated with the response, for different strengths of association *β* (*x*-axis). When *β* = 0, the value of the curve is the estimated Type I error probability. The targeted Type I error rate was 0.05. When *β* ≠ 0, the value of the curve is the power of the hypothesis test. Better methods have a Type I error rate at most 0.05, and a power curve above other methods. Exposure-response functions used are linear, nonlinear, linear interaction, and sinusoid (nonlinear interaction).

**Figure 2.**
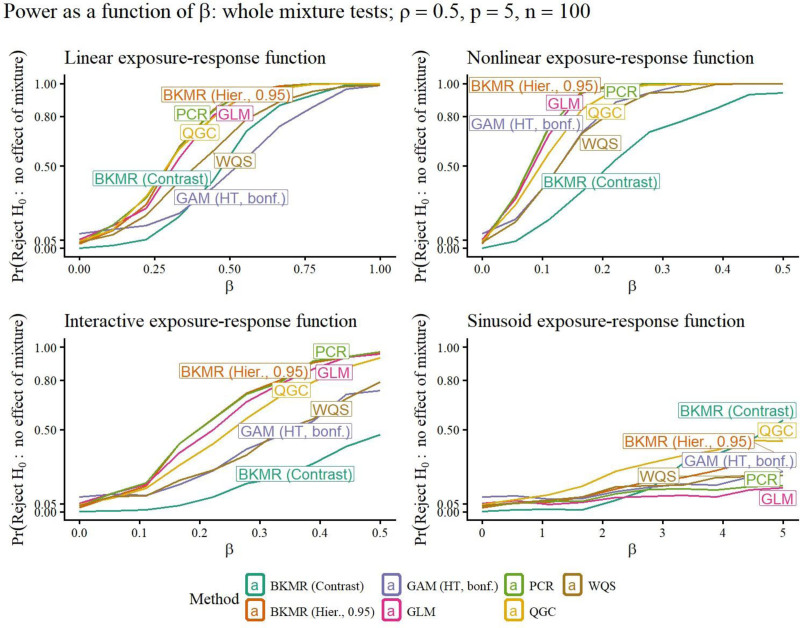
For each method (curve), the y-value is the probability of rejecting *H*_0_: the overall mixture is not associated with the response, for different strengths of association *β* (*x*-axis). When *β* = 0, the value of the curve is the estimated Type I error probability. The targeted Type I error rate was 0.05. When *β* ≠ 0, the value of the curve is the power of the hypothesis test. Better methods have a Type I error rate at most 0.05, and a power curve above other methods. Exposure-response functions used are linear, nonlinear, linear interaction, and sinusoid (nonlinear interaction).

**Figure 3.**
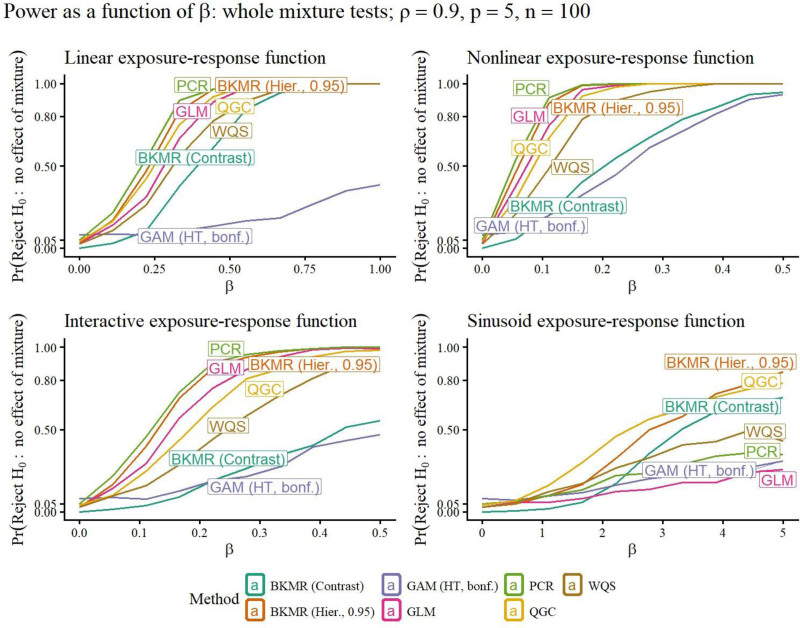
For each method (curve), the y-value is the probability of rejecting *H*_0_: the overall mixture is not associated with the response, for different strengths of association *β* (*x*-axis). When *β* = 0, the value of the curve is the estimated Type I error probability. The targeted Type I error rate was 0.05. When *β* ≠ 0, the value of the curve is the power of the hypothesis test. Better methods have a Type I error rate at most 0.05, and a power curve above other methods. Exposure-response functions used are linear, nonlinear, linear interaction, and sinusoid (nonlinear interaction).

**Figure 4.**
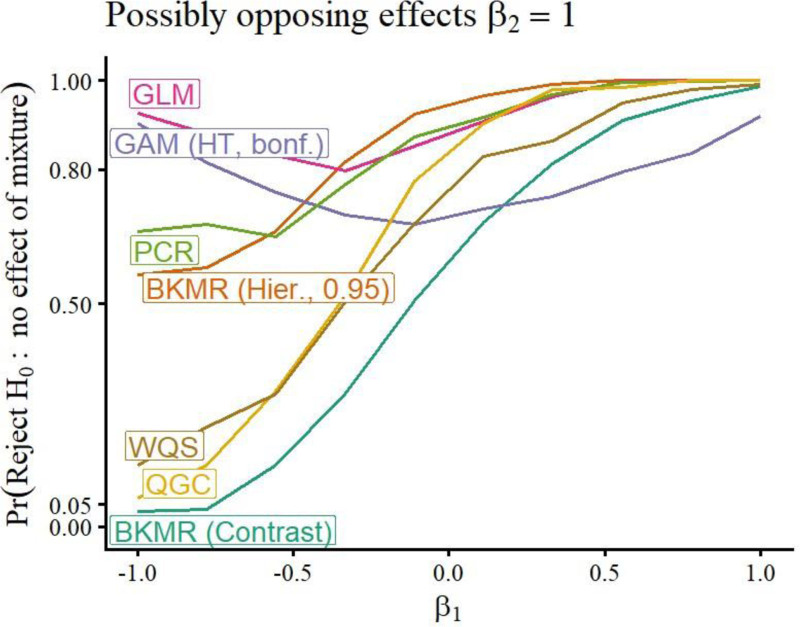
Power curves for whole-mixture hypothesis tests for *n* = 100*, p* = 10*, ρ* = 0.5, possibly opposing effects. For each method (curve), the y-value is the probability of rejecting *H*_0_: the overall mixture is not associated with the response, for different strengths of association *β* (*x*-axis). The exposure-response function used is linear, with possibly opposing effects of mixture components. Note that the *x*-axis endpoints have changed as *E*(*y*|*a*_1_*,a*_2_) = *β*_1_*a*_1_ + *β*_2_*a*_2_, and *β*_2_ is fixed at 1, and *β*_1_ now varies from −1 to 1. Therefore, better methods have all values of their power curve above other methods’.

**Figure 5.**
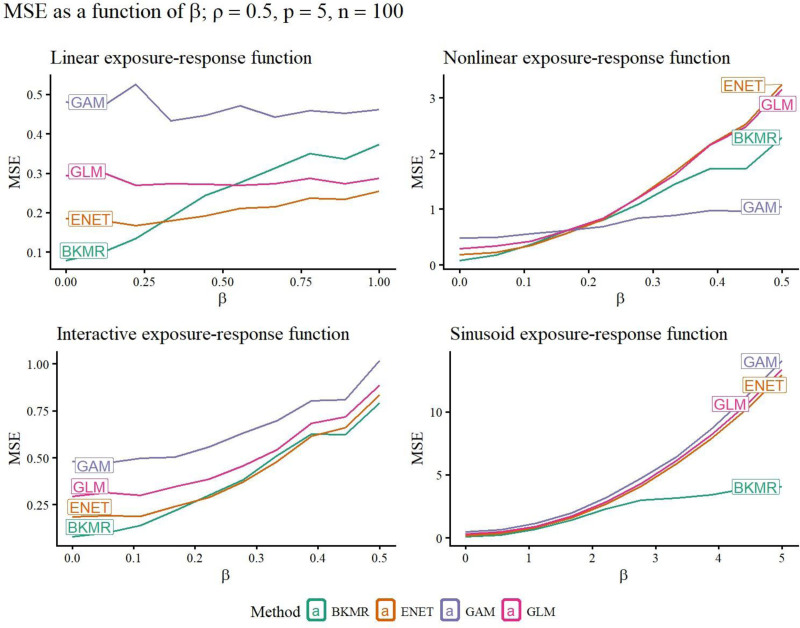
For each method (curve), the y-value is the prediction mean squared error (MSE) on new data, for different strengths of association *β* (*x*-axis). Better methods have lower MSE curves. The new data are generated from the same exposure-response functions as the data used to fit the model, but have no random variation added. Exposure-response functions used are linear, nonlinear, linear interaction, and sinusoid (nonlinear interaction).

The power curves for component-wise hypothesis tests in Figure 1 show that elastic net and BKMR, using a PIP cutoff of 0.50 to determine significance, and GAMs using variable selection, abbreviated as GAM (VS), were over-sensitive, resulting in Type I error rates well above 0.05. GAMs using frequentist hypothesis testing, abbreviated as GAM (HT), GLMs, and BKMR using a PIP cutoff of 0.95 had Type I error rate at or below the nominal value of 0.05. For this reason, in the rest of the paper, we use “GAMs” to refer to GAMs using hypothesis testing, as those results are of primary interest when considering methods that control Type I error rate. GAMs and GLMs had similar performance in the linear, nonlinear, and interactive scenarios, with greater power than BKMR using a PIP cutoff of 0.95. In the sinusoidal scenario, however, BKMR using a PIP cutoff of 0.95 strongly outperformed GAMs and GLMs in power, reflecting the more flexible model’s ability to estimate the complicated exposure-response function. The prediction MSE curves in Figure 5 show generally that more accurately-specified models performed better on prediction MSE when the association between mixture and response is strong.

### Mixture component identification

GAMs and GLMs worked best in many situations for identifying important components of the mixture, with a nominal Type I error rate and competitive power. This was true when misspecified due to nonlinearity (in the case of GLMs) or linear interaction, but was not true for the most complicated, sinusoidal, exposure-response relationship (Figure 1). When the form of the interaction was most complicated in the sinusoidal scenario, only BKMR was easily able to detect the association.

The favorable results for GLMs (specified as linear models) are in part due to the simulation setup. The nonlinear relationships were monotonically increasing, so a misspecified linear model will fit an increasing trend to the data. Our nonlinear and linear interaction scenarios were chosen to represent exposure-response functions that were more complicated than linear models but still relatively simple.

Elastic net, GAMs with variable selection, and BKMR with a PIP cutoff of 0.5 had high Type I error rates that would generally be unacceptable for hypothesis testing, but may be useful for more exploratory analyses. BKMR using the higher cutoff of 0.95 had acceptable Type I error rate, although lower power than other methods in simpler scenarios.

### Whole-mixture tests

The GLM *F*-test is suitable for moderate *p* and *ρ*, but loses power compared with methods that perform whole-mixture tests that use fewer degrees of freedom, such as QGC, WQS, and PCR with higher *p* or *ρ*. Figures 2 and 3, top-left panels, illustrate that QGC and PCR outperform GLMs with increased *ρ* in the linear scenario; the effect of increasing *p* and/or *ρ* can be further seen in Figures S9–S14; https://links.lww.com/EE/A398 in the Supplemental Materials, and we discuss how to interpret these results in the context of applied data analyses in Section “Discussion.” When associations went in opposite directions, QGC, WQS, PCR, and BKMR’s whole-mixture tests lost power, with the loss for QGC, WQS, and BKMR’s contrast test particularly severe (Figure 4). Power for WQS regression was generally lower than QGC, due at least in part to sample splitting used by WQS. In the main simulation scenarios where *p* = 5, GAMs using the Bonferroni correction worked well when *ρ* = 0 (Figure 2), but had uncompetitive power when *ρ* = 0.9 (Figure 3); however, in the opposite-effects scenario, power was still greater than methods other than GLMs (Figure 4). When *p* = 10, increasing *ρ* dramatically decreased GAMs’ power more dramatically when the Bonferroni correction was applied (Figures S12–S14; https://links.lww.com/EE/A398).

BKMR’s default hierarchical variable selection prior resulted in group PIPs that were essentially always greater than 0.50 even under the null hypothesis, displayed in Figure S29; https://links.lww.com/EE/A398. However, when the cutoff for significance was 0.95, empirical performance for hierarchical variable selection was strong in most scenarios, with some power lost in the opposite effects scenario. The BKMR contrast test had appropriate Type I error rate control, but low power compared with parametric methods in the simpler scenarios.

### Prediction

Prediction MSE favored the correctly specified model when the strength of association between the exposures and response, controlled by *β*, was higher. In the linear scenarios, GLM and elastic net were most favored; in the nonlinear scenarios, GAMs were favored, followed closely by BKMR; and in the linear interaction and sinusoidal scenarios, BKMR was consistently best (Figure 5 is representative, with full results in Figures S19–S27; https://links.lww.com/EE/A398). When *β* was low, elastic net and BKMR were best, even in some scenarios where other models were either better-specified or more simply specified. This is likely due to their variable selection procedures removing irrelevant variables from the model, which seems most important for low *β*.

## Discussion

In our simulated scenarios where the number of exposures was moderate (in our simulations, *p* = 5), the correlation between exposures was moderate (in our simulations, *ρ* = 0.5), and the exposure-response function was relatively simple (i.e., not the sinusoid function in our simulations), general methods outperformed novel chemical mixture methods in hypothesis testing. Novel chemical mixture methods should therefore not be a default choice. The choice of model and method should be guided by the researcher’s knowledge of the data and their goals, but the tradeoffs of methods in different scenarios may not have been well-understood to this point due to the lack of simulation studies comparing general methods and novel chemical mixture methods across a range of scenarios. Unless there is something particularly difficult about analyzing a particular data set involving chemical mixture exposures, general methods such as GLMs and GAMs will likely be better choices for the analysis than a more specialized method.

Previous simulation studies have not evaluated the performance of simpler methods applied to simpler (e.g., additive and linear) data-generating processes. Hao et al considers 11 methods, comprised of variants of elastic net/LASSO regression, BKMR, random forests,^19^ and QGC, WQS regression, selection of nonlinear interactions using forward selection (SNIF),^20^ and an ensemble method.^21^ Hao et al recommended SNIF for identifying important mixture components when Type I error rate control was of primary interest.^9^ Lazarevic et al considered six nonlinear regression methods that perform variable selection, and compared them primarily using the F1-statistic, which balances Type I and II error rates. Lazarevic et al found that several nonlinear regression methods were approximately equally suitable for identifying important mixture components, including BKMR using a PIP cutoff of 0.50 to determine the importance of mixture components.^8^ Hoskovec et al compared an interactive Bayesian linear regression model with nonparametric shrinkage,^22^ a clustering model,^23^ BKMR, and linear regression using frequentist hypothesis tests, but examined only interactive data-generating processes. Hoskovec et al recommended the interactive Bayesian linear model using the shrinkage prior when identifying important components was of primary interest and the truth was approximately linear.^7,22^ In contrast to these previous studies, we clearly identify scenarios where simpler methods like additive GLMs (e.g., linear regression), or GAMs with an additive specification, provide more power than more complicated or novel methods while controlling the Type I error rate to a chosen, nominal level.

Our results show that novel methods for chemical mixtures have advantages in hypothesis testing performance when there is high correlation (in our simulations, *ρ* = 0.9) between exposures or many (in our simulations, *p* = 10) moderately correlated exposures (QGC and WQS); or the dose-response function is very nonlinear and interactive (BKMR). There is no clear dividing line between scenarios that allows a definitive, a priori best choice of method for a particular data set. Considering linear (on the scale of the link function *g*) exposure-response relationships, in determining what method might be best, there is interplay between the number of exposure variables, their correlations, the strength of association between the exposures and the response, and the sample size. More variables and higher correlations will tend to reduce the performance of GLMs, while a higher sample size and/or strength of association will increase the performance of GLMs. For a data set with many highly correlated exposure variables and a smaller sample size, QGC or WQS may be appropriate as GLMs may not be able to discern individual associations of the correlated exposures with the response, but if a much larger sample size is obtained, GLMs may then perform better, negating the need for a more specialized method. It can also be difficult to determine whether the true exposure-response function is complicated enough to warrant using BKMR, especially if the overall strength of association is not high. Predictions can be obtained on the data set using different models, using cross-validation to avoid overfitting, to compare the predictive accuracy of each model for those data.^10^ If BKMR or another flexible method provides notably better predictive accuracy than a simpler model, then the more flexible model may make more sense; standard GLM diagnostic plots may suggest model changes that achieve the same goal, however.

Our simulation design was meant to represent aspects of real data sets researchers may find themselves analyzing. For example, although the sample sizes of our simulations were limited to 100 and 400, and the number of exposures was 5, 10, or 20, results from these combinations should represent, roughly, results from other data sets with similar ratios of number of exposures to sample size. Our results for *p* = 5 and *n* = 100, for example, we believe are representative of results that could be expected of other data sets where *p/n* ≈ 0.05. Similarly, although we considered the strength of association controlled by the regression parameter *β* in simulations, with variance of the i.i.d. error terms fixed at 4, each result is likely representative of other scenarios where the signal-to-noise ratio *V ar*(*h*(**a**;*β*))*/V ar*(*ϵ*_*i*_) is preserved with other choices of parameters. However, the simulations do not capture a problematic scenario for PCR, when important components of the mixture are not included in selected PCs. There are certainly many other real-life scenarios not represented in our simulation. For example, with extremely large *n*, most methods will have adequate power to detect practically significant exposure-response association, and the primary factor in choosing a method may then be interpretability with regard to specific research questions.

There are important tradeoffs to using each of the methods. In general, to address correlation between exposures, their associations with the response are combined in some way in the novel methods and PCR. Although only WQS makes the explicit “directional homogeneity” assumption, WQS, QGC, PCR, and BKMR’s whole-mixture tests lost power when effects went in opposite directions in our simulations. Without directional homogeneity, then GLMs or GAMs are likely preferable.

Both WQS and QGC require quantization of continuous exposure variables. This is also sometimes performed with other methods such as GLMs. This can help model nonlinear relationships between the exposures and response and reduce influence of outliers. Quantization loses information from the exposure data.^6^ Near the values of sample quantiles, it can create large jumps in values in the independent variable *q*_*ij*_, when in reality there was a small difference between the original *a*_*ij*_ values, or within one quantile, very different values of *a*_*ij*_ may be given the same value of *q*_*ij*_. The locations of the changes in value are essentially arbitrary. Quantizing continuous exposure variables should therefore be done with caution, and when possible, other methods to model nonlinearity should be preferred.

In Gennings, QGC was criticized for misunderstanding one goal of WQS: to estimate the effects of many small exposures together.^24^ In our “dense” scenarios, capturing this situation, WQS had modestly worse power than QGC and several other methods (Table S17; https://links.lww.com/EE/A398 and Figure S18; https://links.lww.com/EE/A398). This was likely due in part to the sample-splitting used by WQS. If many small effects exist, if they are all in the same direction, our results showed QGC to be the most appropriate choice. Yu et al also note that WQS regression lacks interpretability since *β*_1_ does not have an easily expressed meaning.^6^ In both WQS and QGC the index is a linear combination of the exposures.

BKMR’s PIPs require careful interpretation. Using 0.50 as the cutoff for statistical significance led to high Type I error rates in our simulation. With hierarchical variable selection, group PIPs were nearly always above 0.50 even under the null hypothesis of no association of the mixture with the response, when using default package settings (Figure S29; https://links.lww.com/EE/A398). Using a cutoff of 0.95 resulted in Type I error rates less than 0.05 in our simulations, although this cutoff was chosen essentially arbitrarily and may not be appropriate for all situations. PIPs can also be influenced by the prior distributions, and sensitivity to the priors should be checked as recommended in Bobb et al.^2^ For these reasons, it is difficult to interpret the strength of evidence of association indicated by a PIP unless it is close to 1.0.

As indicated by the high PIPs under the null hypothesis when using hierarchical variable selection (Figure S29; https://links.lww.com/EE/A398), prior inclusion probabilities may need to be tuned according to the number of chemicals in the group. While this tuning requirement is a reasonable component of such a grouped variable selection procedure, it is not mentioned in Bobb et al or Bobb et al, and with inappropriate package default settings, raises the possibility of accidental misuse by practitioners.^2,17^ Predictions can be used to explore a fitted BKMR model and draw conclusions from the analysis. Conclusions based on exploration of the model predictions’ posterior distribution should be differentiated from those based on statistical tests that have appropriate control of Type I error rates.

The traditional methods also require tradeoffs. GLMs require a user-specified dose-response function (although this is also true of QGC and WQS). PCR can miss important components of the mixture in the dimension reduction step and also assumes a linear model. GAMs lack a good joint test of association, making them unsuitable when there is too much correlation between exposures. In general, GLMs and GAMs should be used when there are not a large number of exposure variables relative to the sample size, and the correlation between them is moderate.

Finally, many of the methods considered, including general ones, often sacrifice some interpretability, which is important when communicating results to community members or policymakers. GLMs provide excellent interpretability, although there are scenarios in which they are unsuitable. Individual component effects can be read directly from the GLM model output, and mixture effects and confidence intervals for GLMs and GAMs could be estimated using contrasts between different exposure values, such as contrasting predicted values when each component is set to its 0.75 quantile versus its 0.25 quantile. One useful strategy may be to use GLMs to obtain interpretable summaries of results, and check robustness of the overall results to the strong GLM assumptions using a more complicated model such as GAMs, or a novel mixture method, as was done in Rosato et al and Lebeaux et al, for example.^1,25^

## Conclusion

As interest in modeling chemical mixtures continues to grow, more innovative techniques for analyzing these types of data will be proposed. The challenges in this field are significant, and we hope will continue to spur new statistical developments. Novel approaches hold great promise to further our ability to understand the health effects of environmental contaminants or other types of chemical mixtures, and will advance other areas of statistical methodology. Given enthusiasm for new approaches, it is critical to evaluate these over a standard set of criteria to ensure appropriate models and methods can be selected for applied research.

## Conflicts of interest statement

The authors declare that they have no conflicts of interest with regard to the content of this report.

## Supplementary Material
