# Supplementary material for: When are novel methods for analyzing complex chemical mixtures in epidemiology beneficial?
Source: Environ Epidemiol. 2026 Jan 14;10(1):e456. doi: 10.1097/EE9.0000000000000456 (PMC12806588; doi:10.1097/EE9.0000000000000456)
Supplement: Supplementary file 1 [file ee9-10-e456-s001.pdf]

Supplemental Materials for: When are novel methods for analyzing complex chemical mixtures in epidemiology beneficial?

Nate Wiecha, Emily Griffith, Brian J. Reich, Jane A. Hoppin

December 2025

S1 Implementation of methods in the simulation study

S1.1 Methods that test for individual components' association with the response

Two methods of identifying important components of the mixture were used with generalized additive models (GAMs), both implemented in the R package mgcv.[1] Frequentist hypothesis tests were denoted in results tables and figures as "GAM (HT)", and variable selection penalties on the smooth terms were denoted in results tables and figures as "GAM (VS)". For GAMs using frequentist hypothesis tests, smoothing parameters were estimated using restricted maximum likelihood (REML) to allow adjustment for smoothing parameter uncertainty in hypothesis tests as recommended in Wood.[2]

Bayesian kernel machine regression (BKMR),[3] implemented in the R package bkmr,[4] yields posterior inclusion probabilities (PIPs) for each variable. A PIP closer to 1.0 indicates greater importance of the mixture component, while a PIP closer to 0 indicates less importance. PIP cutoffs of 0.5 and 0.95 were both used to determine whether a variable was selected into the

model, i.e., for the 0.5 cutoff, a mixture component was identified as important if its PIP exceeded 0.5. The cutoff of 0.5 was tested because Lazarevic et al. found that a cutoff of 0.5 results in good variable selection performance.[5] The cutoff of 0.95 was motivated by analogy to p-values and target Type I error probability of 0.05, however, PIPs are not designed to be analogous to p-values or have particular frequentist properties.

For BKMR, 10,000 Markov Chain Monte Carlo (MCMC) iterations were used as burn-in and 10,000 iterations used as posterior samples. Package-default priors were used, and convergence of MCMC was checked using trace plots on example runs using simulation settings. Elastic net was implemented using the glmnet R package,[6] with hyperparameters were chosen by 5-fold cross-validation (CV) performed by the caret package.[7] Finally, a generalized linear model (GLM) was used, with identity link function and Gaussian likelihood, testing the null hypothesis $H_0 : \beta_1 = 0$. GAMs did not include interaction terms for computational tractability. Frequentist hypothesis tests for GAMs and GLMs were conducted by rejecting H_0 if the p-value was less than a nominal value of $\alpha = 0.05$.

S1.2 Methods that test for association between the overall mixture and the response

For GAMs using frequentist hypothesis tests, to conduct a whole-mixture test of association, the individual tests' p-values were used with Bonferroni's correction. An alternative approach is to conduct a generalized likelihood ratio test to compare the fit of the intercept-only GAM with that of the full GAM. This is noted in the documentation of mgcv to potentially be inaccurate, and in initial simulations did not control the Type I error with $p = 10$ despite being based on frequentist justification, so was not included in the final results. For BKMR, joint significance was tested by estimating a contrast $\gamma = h\left(Q_1(0.75), \dots, Q_p(0.75)\right) - h\left(Q_1(0.25), \dots, Q_p(0.25)\right)$, where

$Q_j(w)$ is the wth quantile of exposure j, $w \in [0,1]$, $j = 1,\ldots,p$. An approximate test of H_0: $\gamma = 0$ was performed at the $\alpha = 0.05$ level by comparing $\frac{\hat{\gamma}}{SD(\gamma)}$ to a $N(0,1)$ null distribution, where $\hat{\gamma}$ is the posterior mean of γ and $SD(\gamma)$ is the posterior standard deviation of γ. This is an approximate form of a test recommended in Bobb et al.[8] The hierarchical variable selection prior was also used to test joint association, with all exposure variables included in a single group. With hierarchical variable selection, group PIPs were nearly always above 0.50 even under the null hypothesis of no association of the mixture with the response, when using default package settings (Figure S29), so only results for the 0.95 PIP cutoff are presented.

For principal components regression (PCR), enough principal components were selected to explain at least 75% of the exposure variance. An F-test on the estimated regression coefficients was performed to test for an effect of the mixture on the response. For weighted quantile sum (WQS) regression, implemented in the R package gWQS,[9] and quantile g-computation (QGC), implemented in the R package gqcomp,[10] default settings were used. For WQS, 40% of the sample was used to estimate weights and the remaining observations were used for inference, and a positive association between the mixture and response was assumed. As the GLM was used with a Gaussian likelihood, an F-test of joint significance was also used; with other likelihoods other tests of joint significance are available.

S2 Power curves

This section provides all power curves for all scenarios and methods.

S2.1 Individual component hypothesis tests' power curves

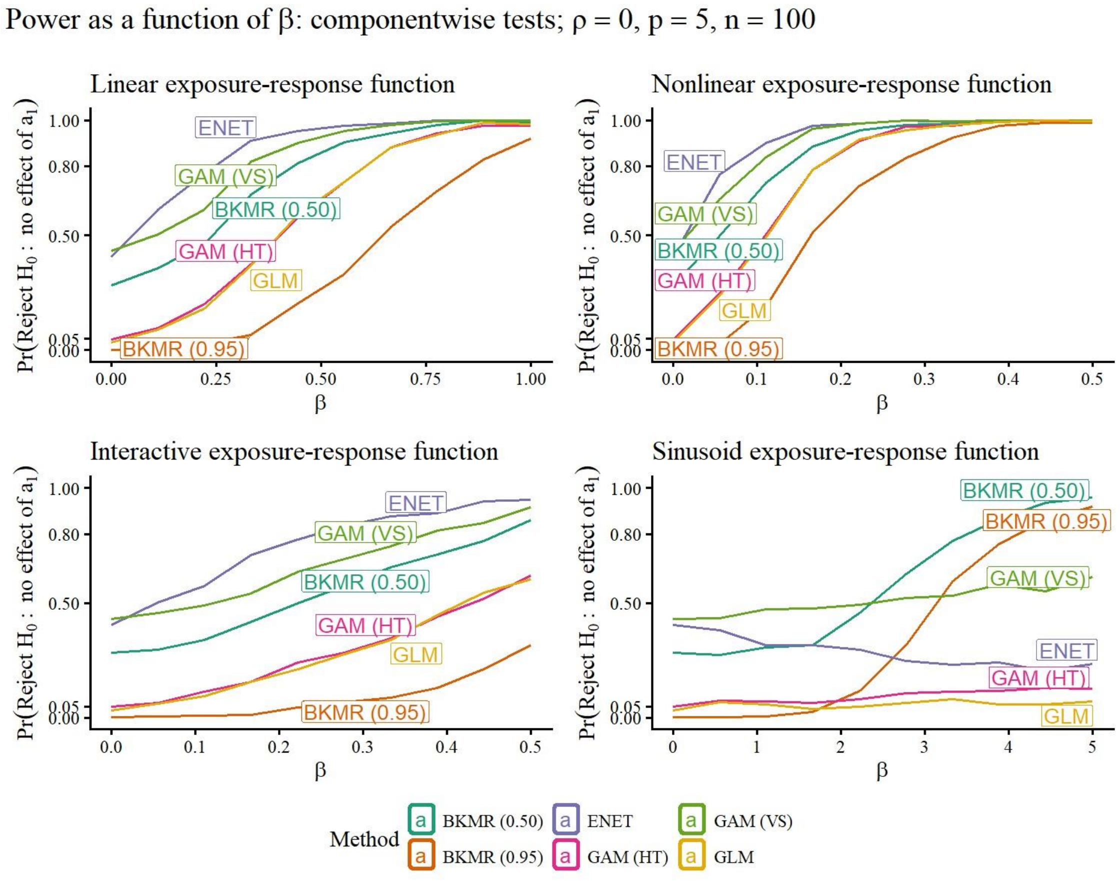

Figure S1: For each method (curve), the y-value is the probability of rejecting H_0: first mixture component a_1 not associated with the response, for different strengths of association β (x-axis). When $\beta = 0$, the value of the curve is the estimated Type I error probability. The targeted Type I error rate was 0.05. When $\beta \neq 0$, the value of the curve is the power of the hypothesis test. Better methods have Type I error rate at most 0.05, and power curve above other methods. Exposure-response functions used are linear, nonlinear, linear interaction, and sinusoid (nonlinear interaction).

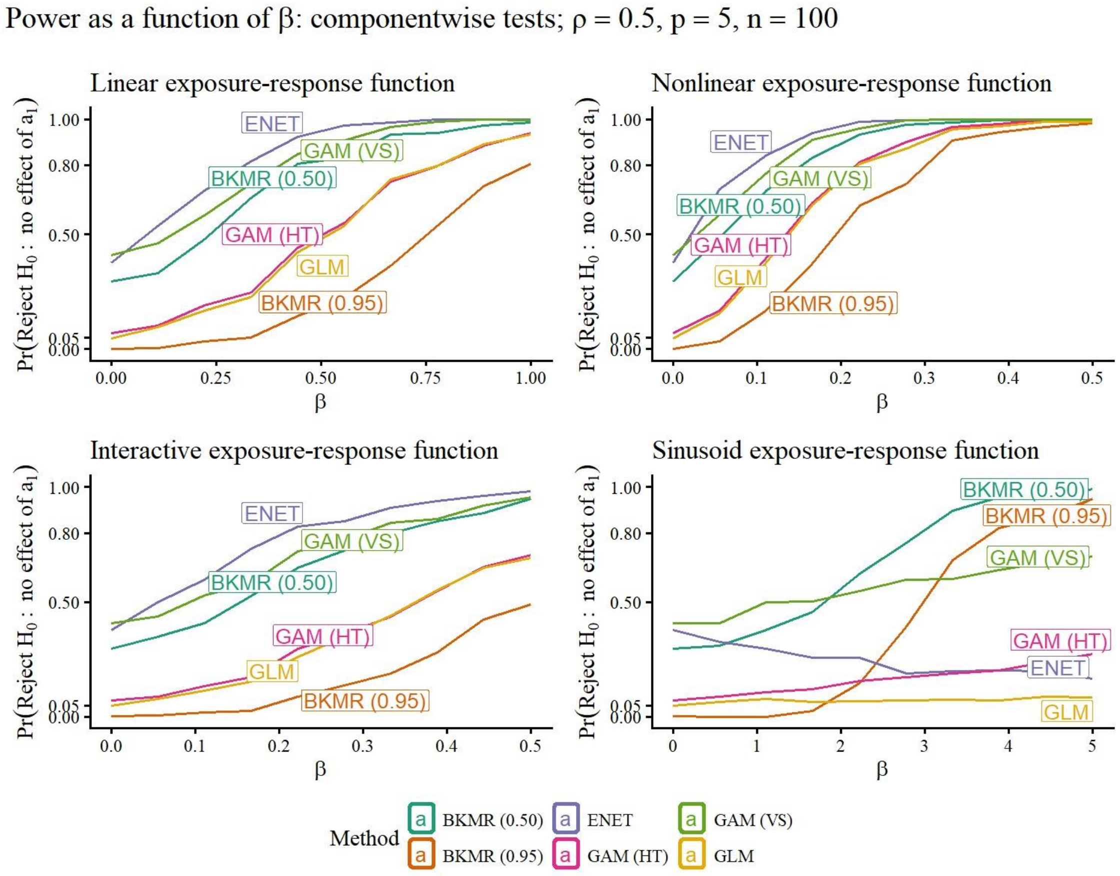

Figure S2: For each method (curve), the y-value is the probability of rejecting H_0: first mixture component a_1 not associated with the response, for different strengths of association β (x-axis). When $\beta = 0$, the value of the curve is the estimated Type I error probability. The targeted Type I error rate was 0.05. When $\beta \neq 0$, the value of the curve is the power of the hypothesis test. Better methods have Type I error rate at most 0.05, and power curve above other methods. Exposure-response functions used are linear, nonlinear, linear interaction, and sinusoid (nonlinear interaction).

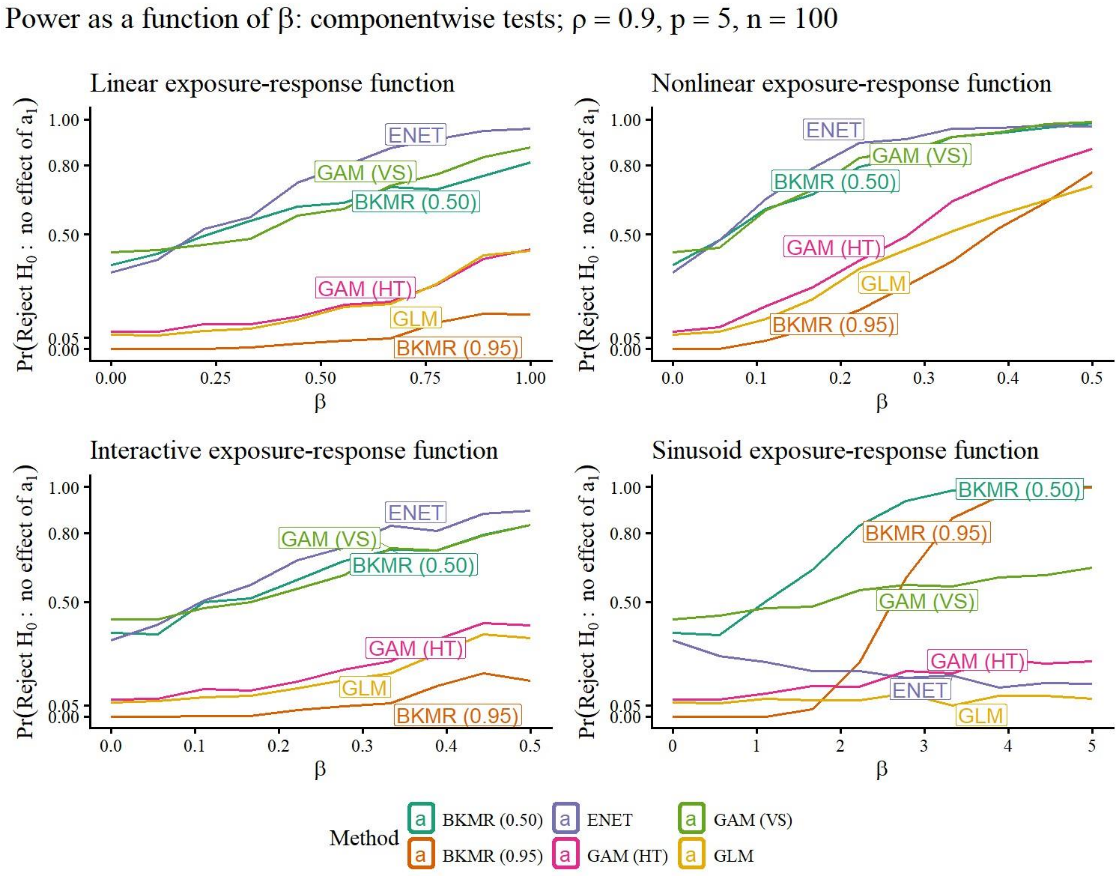

Figure S3: For each method (curve), the y-value is the probability of rejecting H_0: first mixture component a_1 not associated with the response, for different strengths of association β (x-axis). When $\beta = 0$, the value of the curve is the estimated Type I error probability. The targeted Type I error rate was 0.05. When $\beta \neq 0$, the value of the curve is the power of the hypothesis test. Better methods have Type I error rate at most 0.05, and power curve above other methods. Exposure-response functions used are linear, nonlinear, linear interaction, and sinusoid (nonlinear interaction).

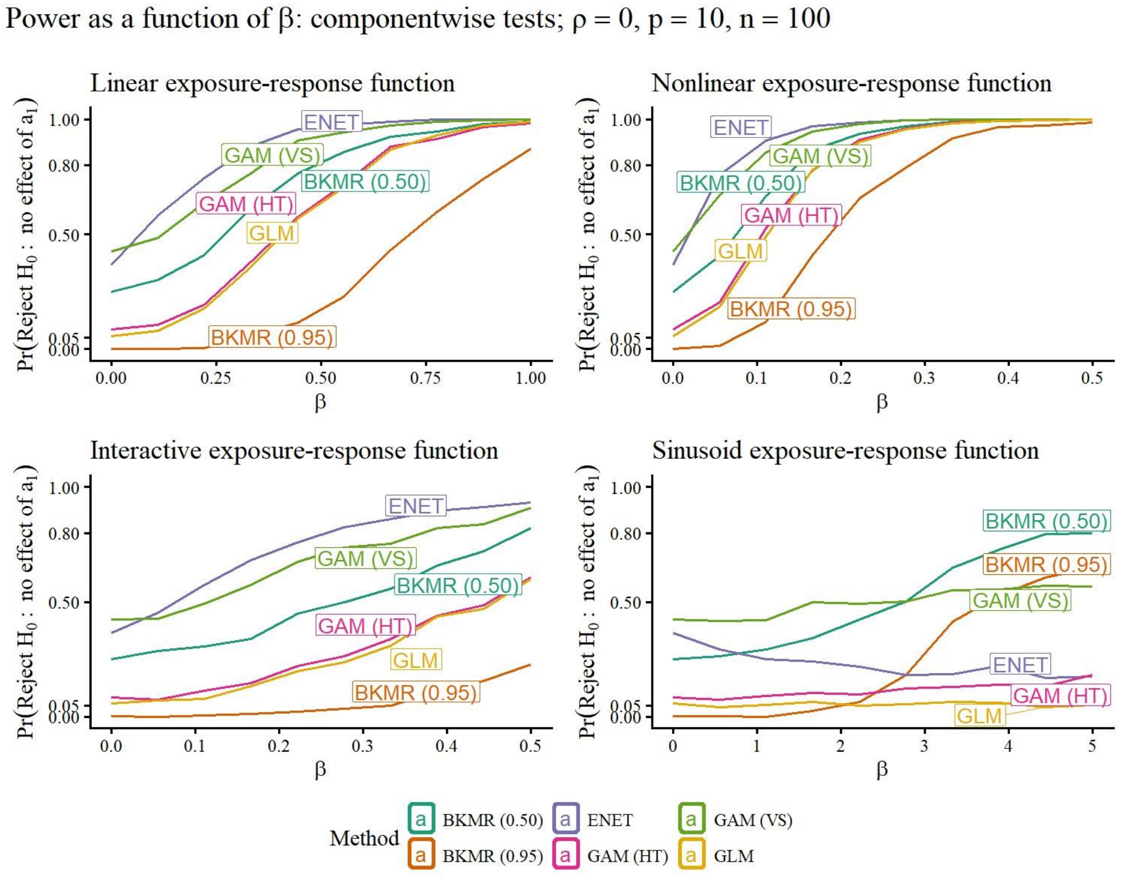

Figure S4: For each method (curve), the y-value is the probability of rejecting H_0: first mixture component a_1 not associated with the response, for different strengths of association β (x-axis). When $\beta = 0$, the value of the curve is the estimated Type I error probability. The targeted Type I error rate was 0.05. When $\beta \neq 0$, the value of the curve is the power of the hypothesis test. Better methods have Type I error rate at most 0.05, and power curve above other methods. Exposure-response functions used are linear, nonlinear, linear interaction, and sinusoid (nonlinear interaction).

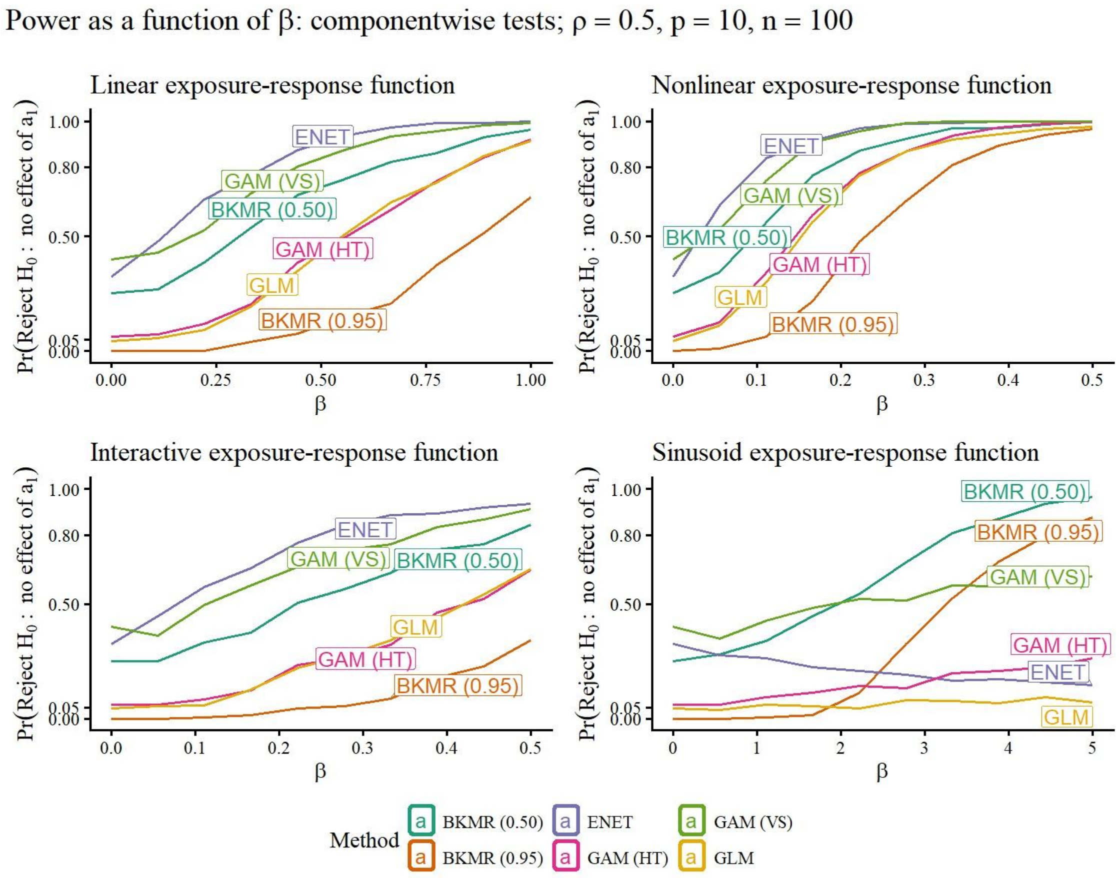

Figure S5: For each method (curve), the y-value is the probability of rejecting H_0: first mixture component a_1 not associated with the response, for different strengths of association β (x-axis). When $\beta = 0$, the value of the curve is the estimated Type I error probability. The targeted Type I error rate was 0.05. When $\beta \neq 0$, the value of the curve is the power of the hypothesis test. Better methods have Type I error rate at most 0.05, and power curve above other methods. Exposure-response functions used are linear, nonlinear, linear interaction, and sinusoid (nonlinear interaction).

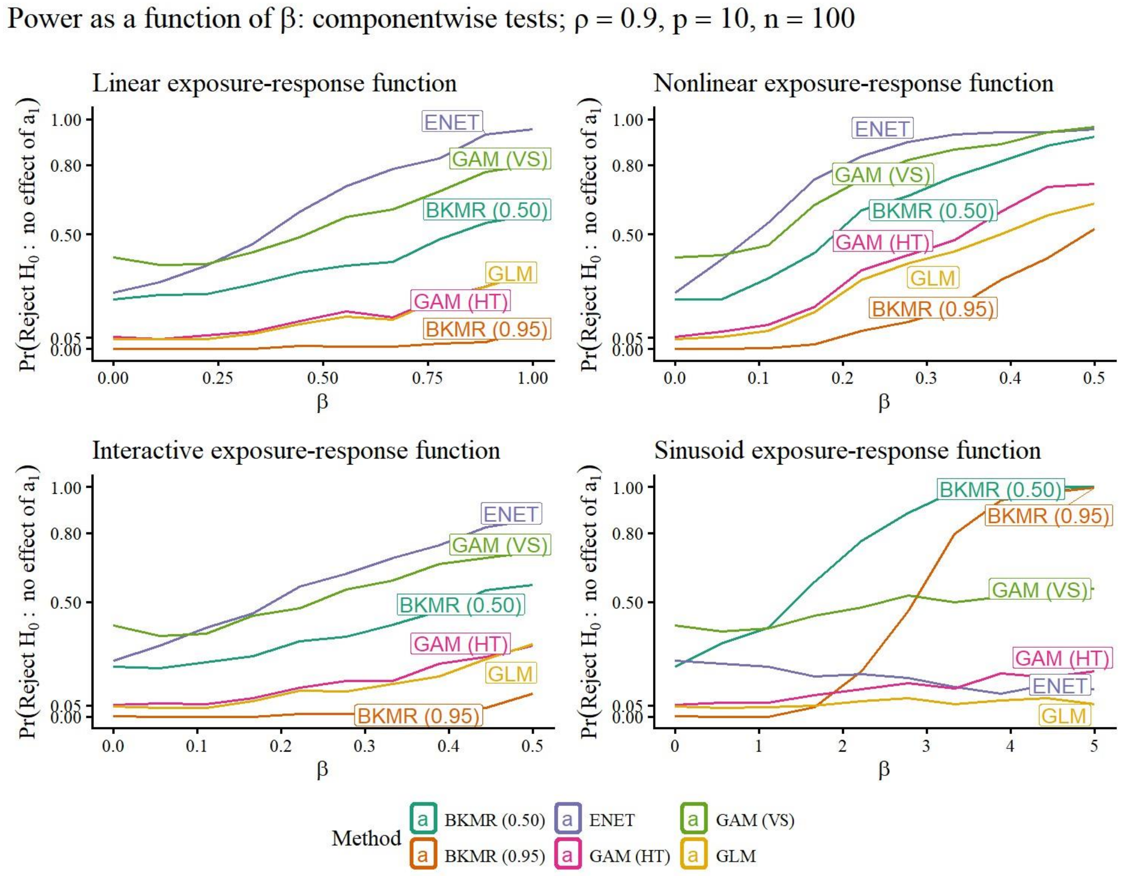

Figure S6: For each method (curve), the y-value is the probability of rejecting H_0: first mixture component a_1 not associated with the response, for different strengths of association β (x-axis). When $\beta = 0$, the value of the curve is the estimated Type I error probability. The targeted Type I error rate was 0.05. When $\beta \neq 0$, the value of the curve is the power of the hypothesis test. Better methods have Type I error rate at most 0.05, and power curve above other methods. Exposure-response functions used are linear, nonlinear, linear interaction, and sinusoid (nonlinear interaction).

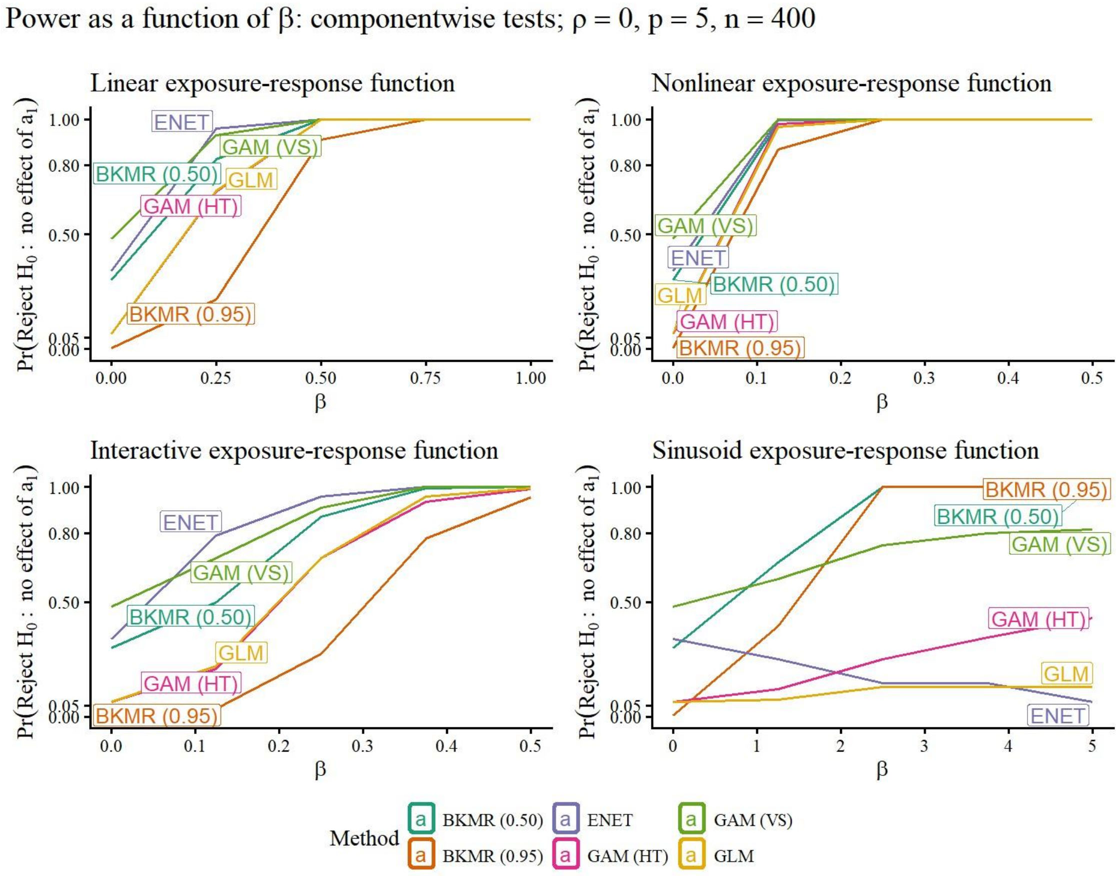

Figure S7: For each method (curve), the y-value is the probability of rejecting H_0: first mixture component a_1 not associated with the response, for different strengths of association β (x-axis). When $\beta = 0$, the value of the curve is the estimated Type I error probability. The targeted Type I error rate was 0.05. When $\beta \neq 0$, the value of the curve is the power of the hypothesis test. Better methods have Type I error rate at most 0.05, and power curve above other methods. Exposure-response functions used are linear, nonlinear, linear interaction, and sinusoid (nonlinear interaction).

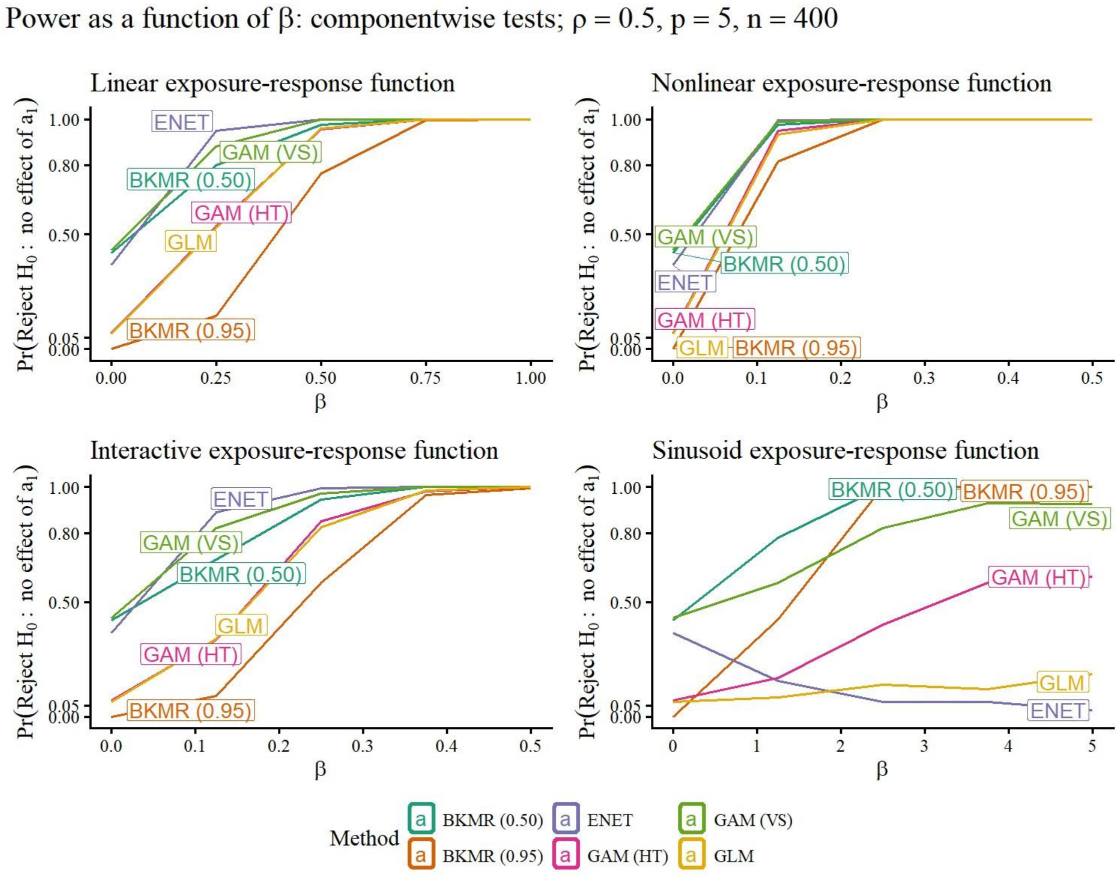

Figure S8: For each method (curve), the y-value is the probability of rejecting H_0: first mixture component a_1 not associated with the response, for different strengths of association β (x-axis). When $\beta = 0$, the value of the curve is the estimated Type I error probability. The targeted Type I error rate was 0.05. When $\beta \neq 0$, the value of the curve is the power of the hypothesis test. Better methods have Type I error rate at most 0.05, and power curve above other methods. Exposure-response functions used are linear, nonlinear, linear interaction, and sinusoid (nonlinear interaction).

S2.2 Whole-mixture hypothesis tests' power curves

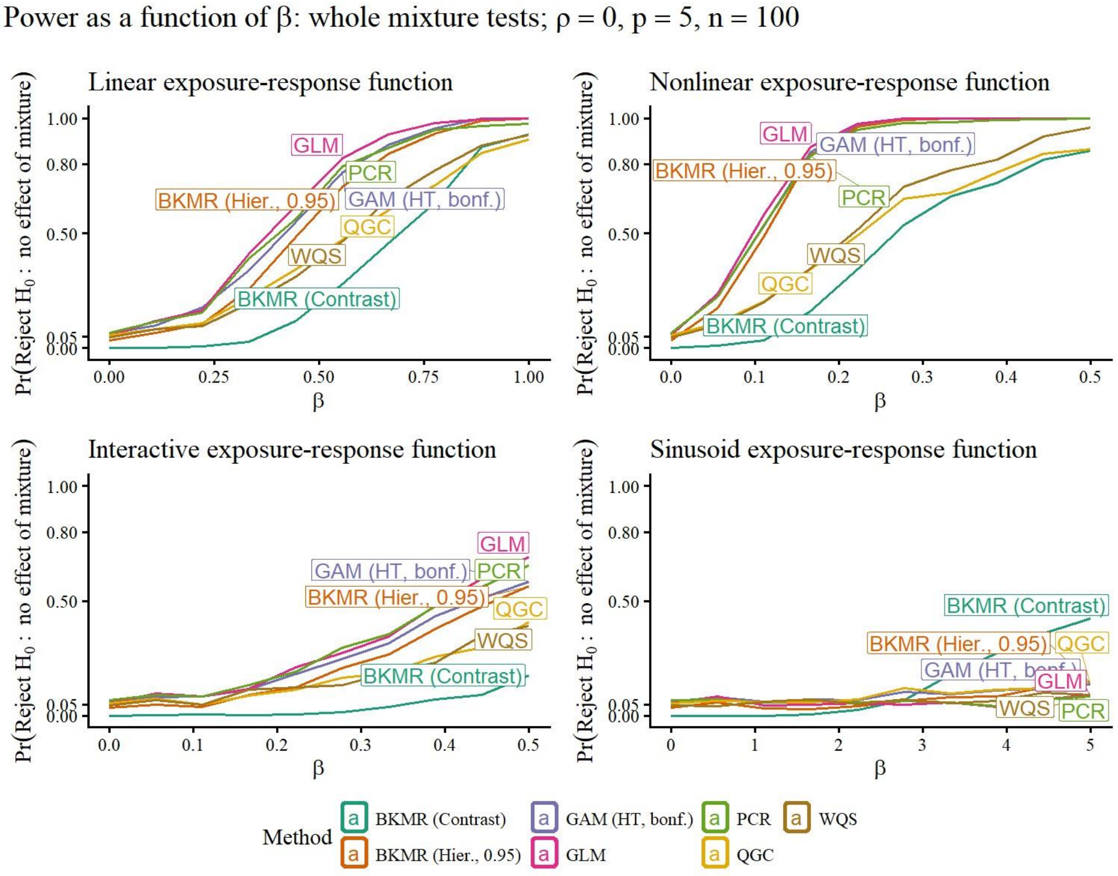

Figure S9: For each method (curve), the y-value is the probability of rejecting H_0: the overall mixture is not associated with the response, for different strengths of association β (x-axis). When $\beta = 0$, the value of the curve is the estimated Type I error probability. The targeted Type I error rate was 0.05. When $\beta \neq 0$, the value of the curve is the power of the hypothesis test. Better methods have Type I error rate at most 0.05, and power curve above other methods. Exposure-response functions used are linear, nonlinear, linear interaction, and sinusoid (nonlinear interaction).

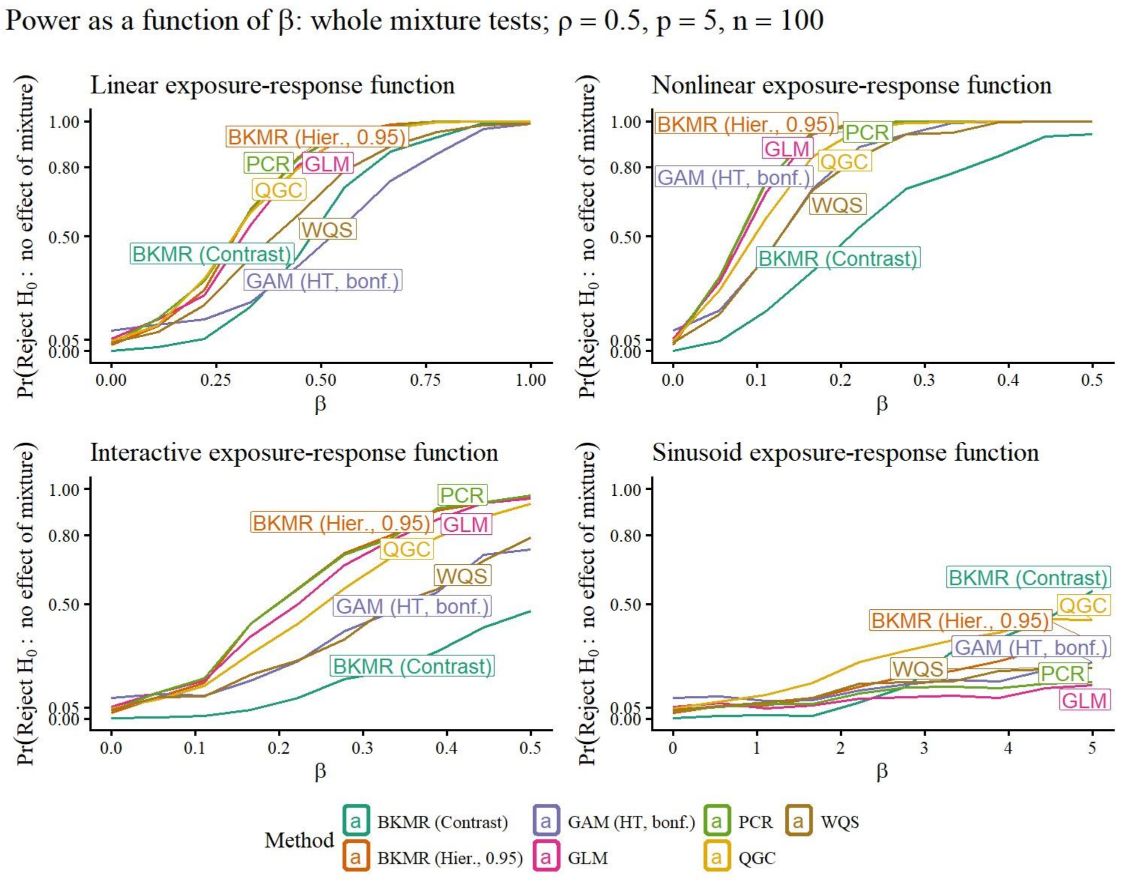

Figure S10: For each method (curve), the y-value is the probability of rejecting H_0: the overall mixture is not associated with the response, for different strengths of association β (x-axis). When $\beta = 0$, the value of the curve is the estimated Type I error probability. The targeted Type I error rate was 0.05. When $\beta \neq 0$, the value of the curve is the power of the hypothesis test. Better methods have Type I error rate at most 0.05, and power curve above other methods. Exposure-response functions used are linear, nonlinear, linear interaction, and sinusoid (nonlinear interaction).

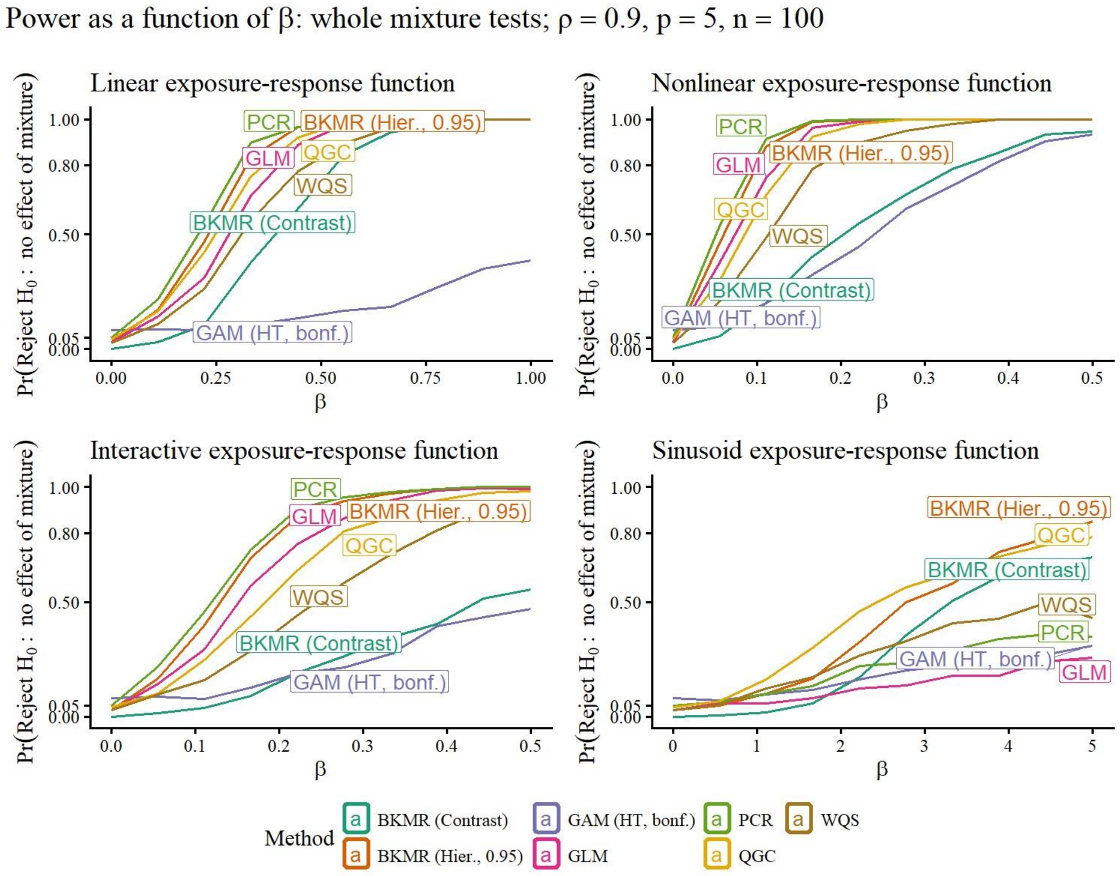

Figure S11: For each method (curve), the y-value is the probability of rejecting H_0: the overall mixture is not associated with the response, for different strengths of association β (x-axis). When $\beta = 0$, the value of the curve is the estimated Type I error probability. The targeted Type I error rate was 0.05. When $\beta \neq 0$, the value of the curve is the power of the hypothesis test. Better methods have Type I error rate at most 0.05, and power curve above other methods. Exposure-response functions used are linear, nonlinear, linear interaction, and sinusoid (nonlinear interaction).

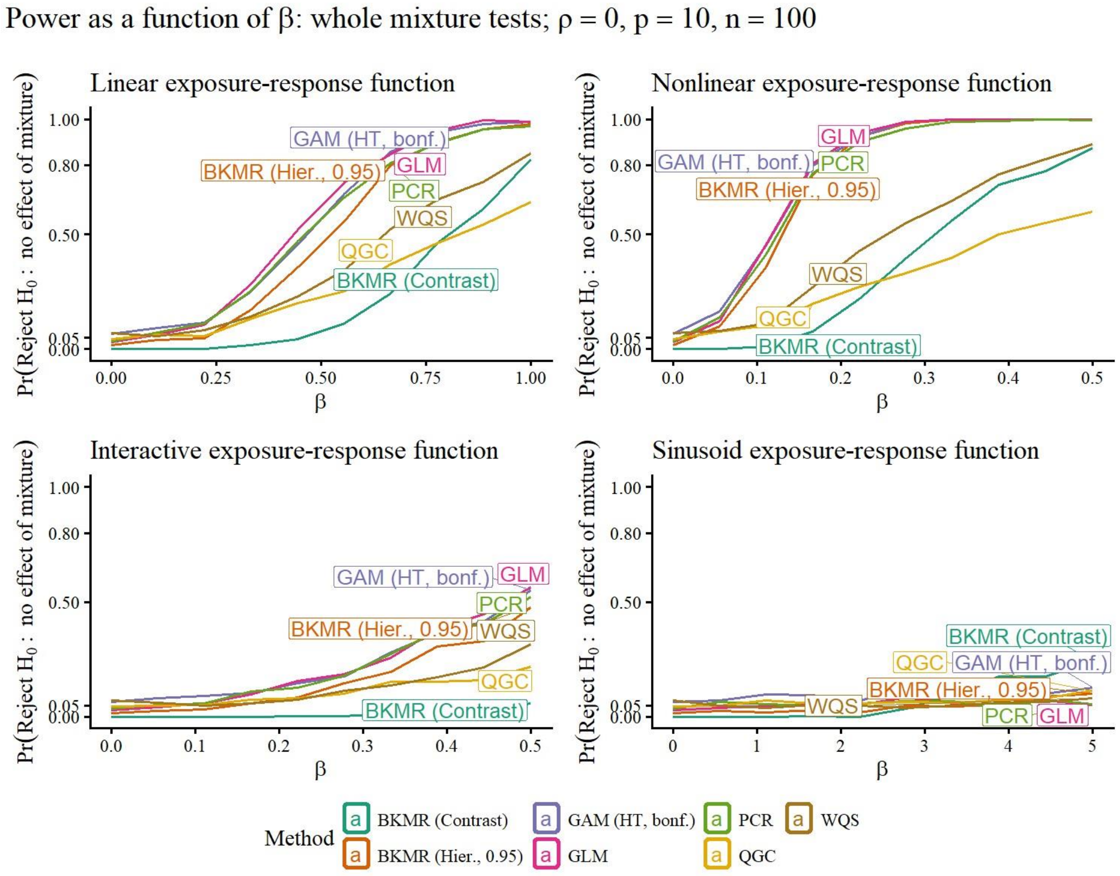

Figure S12: For each method (curve), the y-value is the probability of rejecting H_0: the overall mixture is not associated with the response, for different strengths of association β (x-axis). When $\beta = 0$, the value of the curve is the estimated Type I error probability. The targeted Type I error rate was 0.05. When $\beta \neq 0$, the value of the curve is the power of the hypothesis test. Better methods have Type I error rate at most 0.05, and power curve above other methods. Exposure-response functions used are linear, nonlinear, linear interaction, and sinusoid (nonlinear interaction).

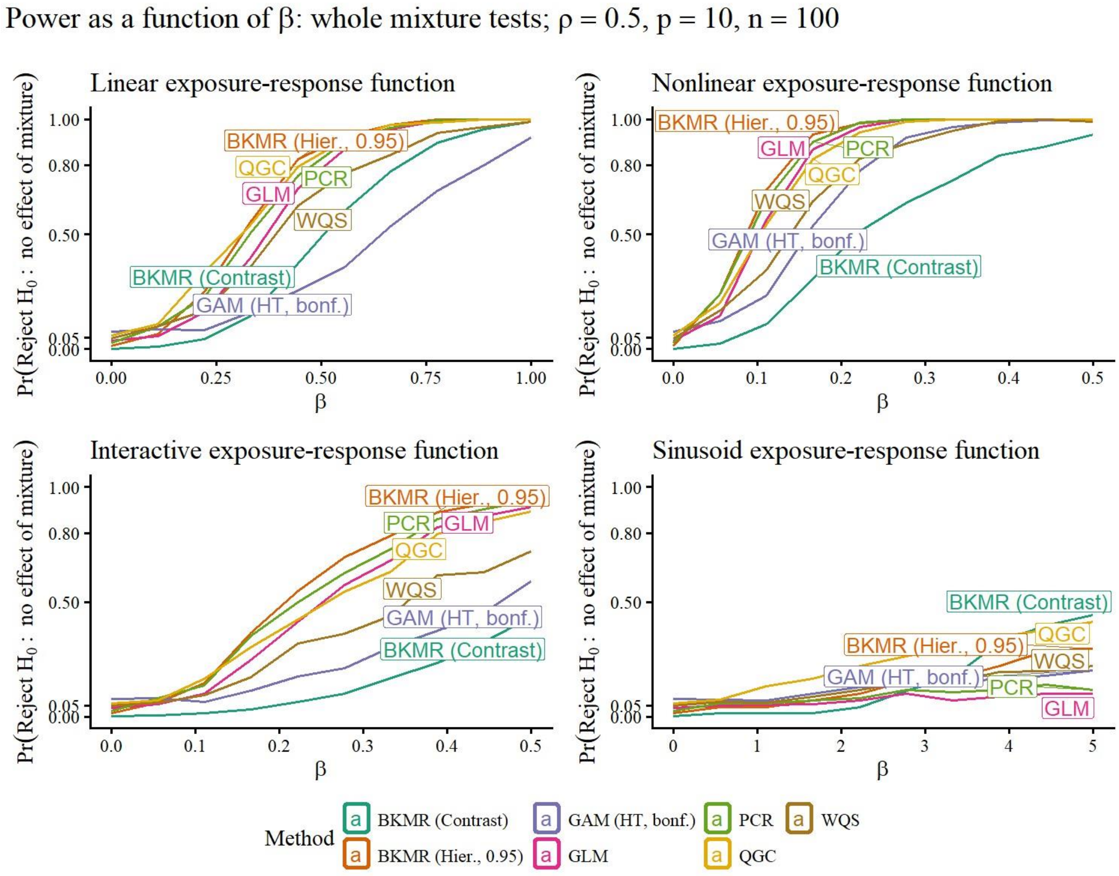

Figure S13: For each method (curve), the y-value is the probability of rejecting H_0: the overall mixture is not associated with the response, for different strengths of association β (x-axis). When $\beta = 0$, the value of the curve is the estimated Type I error probability. The targeted Type I error rate was 0.05. When $\beta \neq 0$, the value of the curve is the power of the hypothesis test. Better methods have Type I error rate at most 0.05, and power curve above other methods. Exposure-response functions used are linear, nonlinear, linear interaction, and sinusoid (nonlinear interaction).

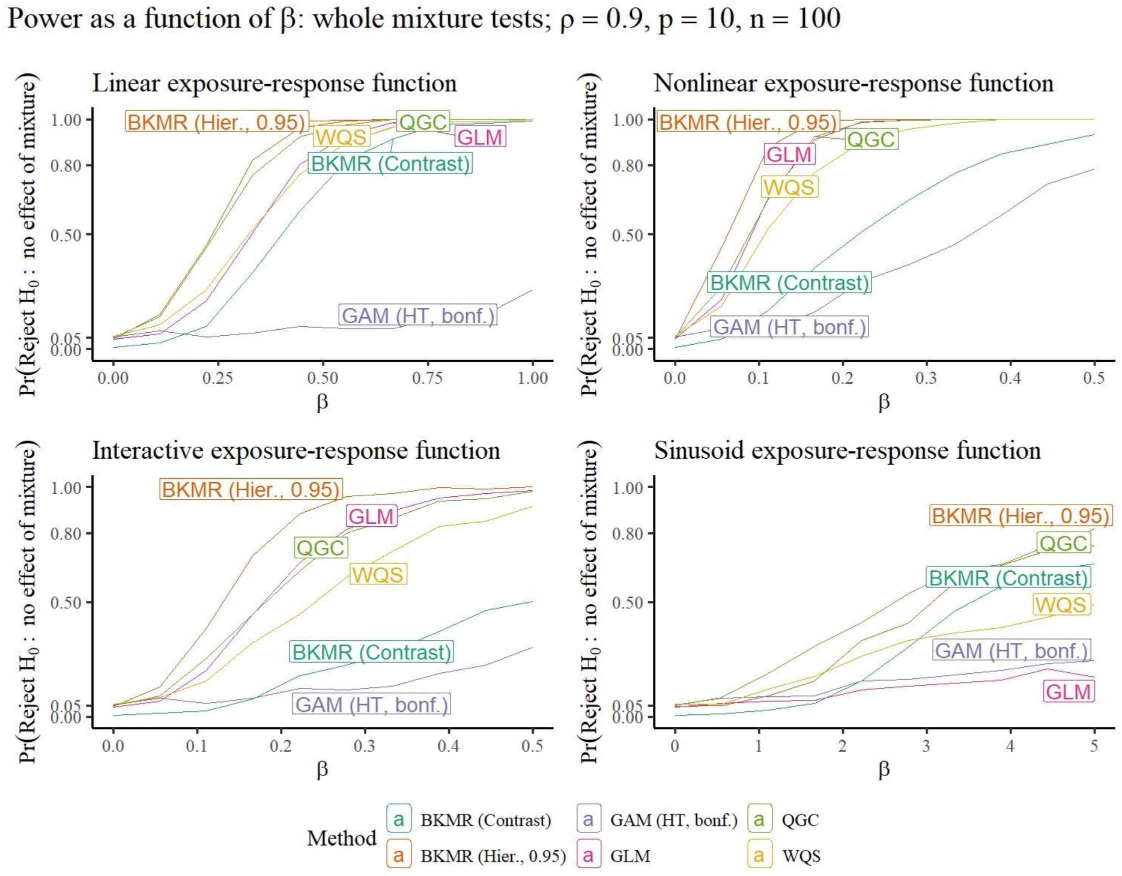

Figure S14: For each method (curve), the y-value is the probability of rejecting H_0: the overall mixture is not associated with the response, for different strengths of association β (x-axis). When $\beta = 0$, the value of the curve is the estimated Type I error probability. The targeted Type I error rate was 0.05. When $\beta \neq 0$, the value of the curve is the power of the hypothesis test. Better methods have Type I error rate at most 0.05, and power curve above other methods. Exposure-response functions used are linear, nonlinear, linear interaction, and sinusoid (nonlinear interaction).

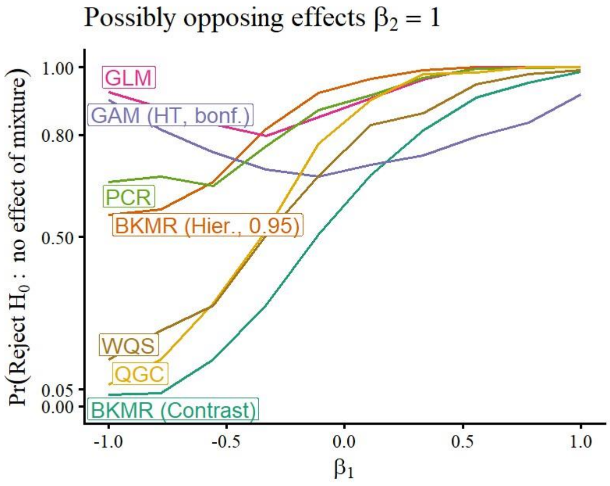

Figure S15: Power curves for whole-mixture hypothesis tests for n = 100, p = 10, ρ = 0.5, possibly opposing effects. For each method (curve), the y-value is the probability of rejecting H_0: the overall mixture is not associated with the response, for different strengths of association β (x-axis). Exposure-response function used is linear with possibly opposing effects of mixture components. Note that the x-axis endpoints have changed as $E(y_i|a_{1i},a_{2i})$ = $\beta_1 a_1$ + $\beta_2 a_2$, and β_2 is fixed at 1, and β_1 now varies from -1 to 1. Therefore better methods have all values of their power curve above other methods'.

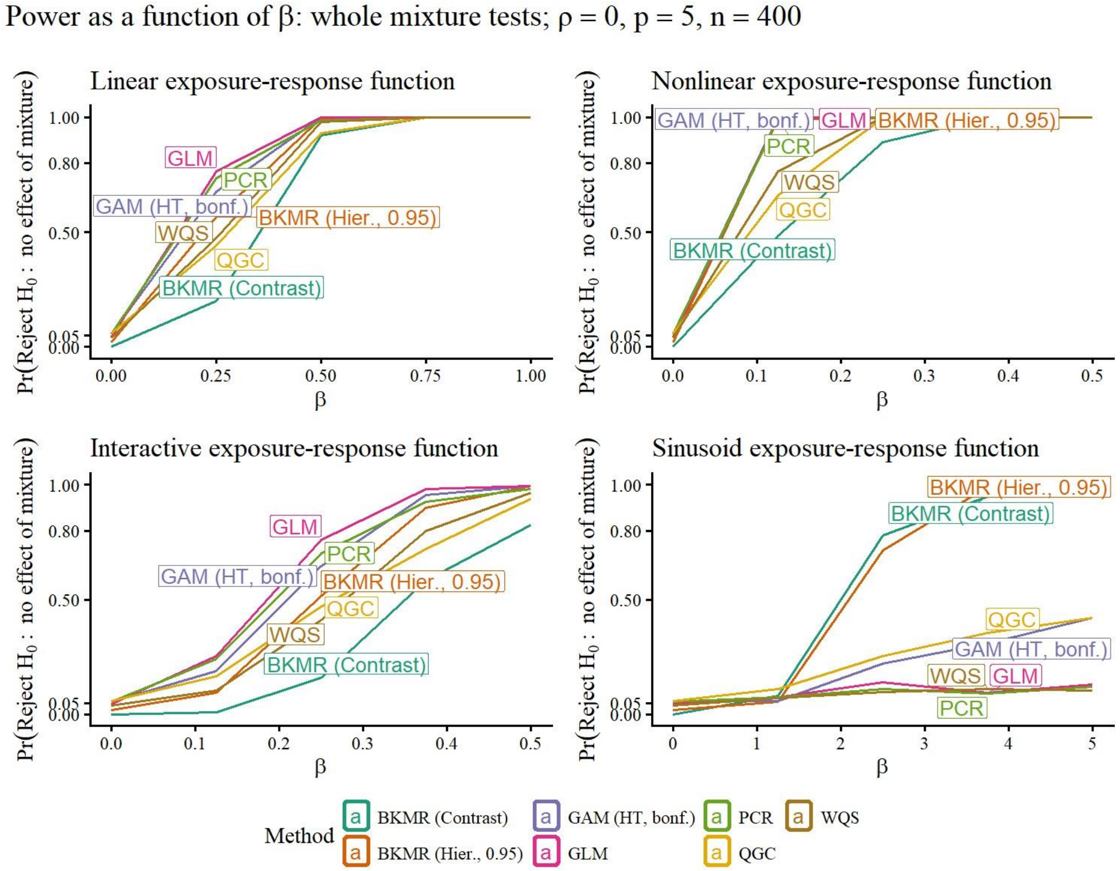

Figure S16: For each method (curve), the y-value is the probability of rejecting H_0: the overall mixture is not associated with the response, for different strengths of association β (x-axis). When $\beta = 0$, the value of the curve is the estimated Type I error probability. The targeted Type I error rate was 0.05. When $\beta \neq 0$, the value of the curve is the power of the hypothesis test. Better methods have Type I error rate at most 0.05, and power curve above other methods. Exposure-response functions used are linear, nonlinear, linear interaction, and sinusoid (nonlinear interaction).

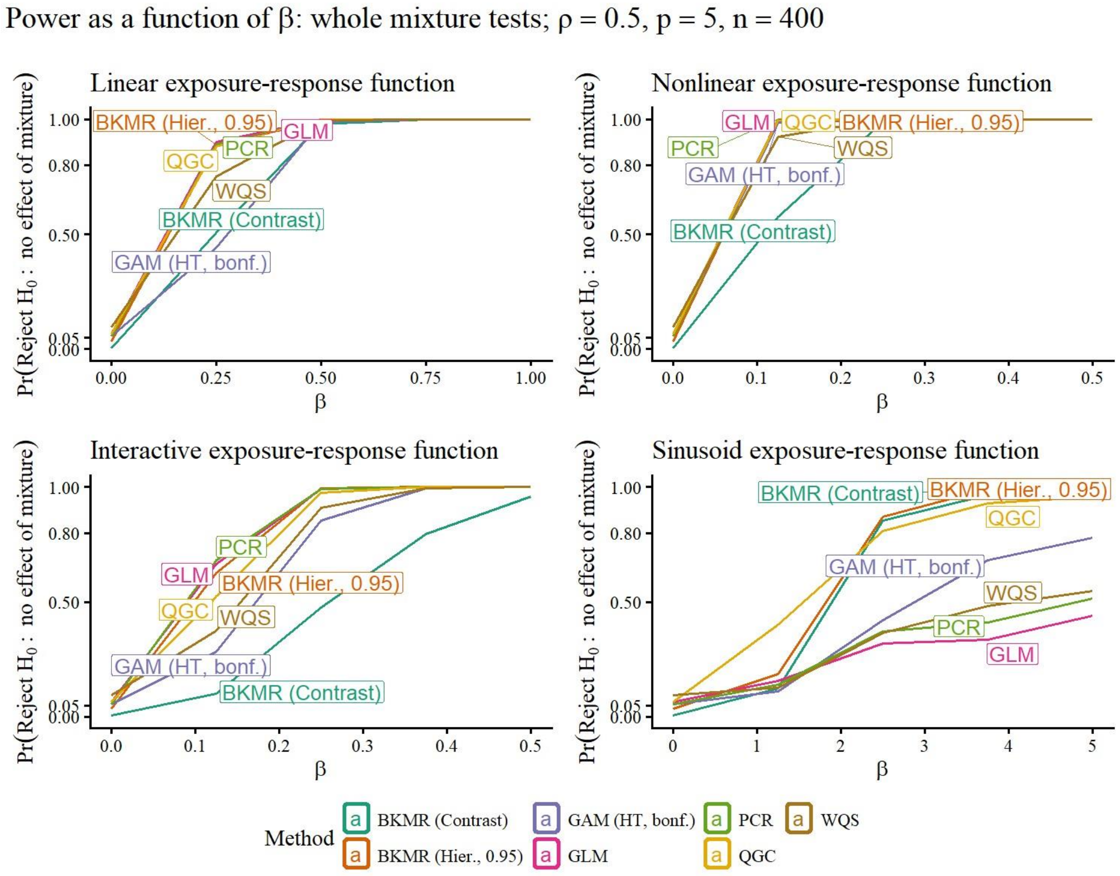

Figure S17: For each method (curve), the y-value is the probability of rejecting H_0: the overall mixture is not associated with the response, for different strengths of association β (x-axis). When $\beta = 0$, the value of the curve is the estimated Type I error probability. The targeted Type I error rate was 0.05. When $\beta \neq 0$, the value of the curve is the power of the hypothesis test. Better methods have Type I error rate at most 0.05, and power curve above other methods. Exposure-response functions used are linear, nonlinear, linear interaction, and sinusoid (nonlinear interaction).

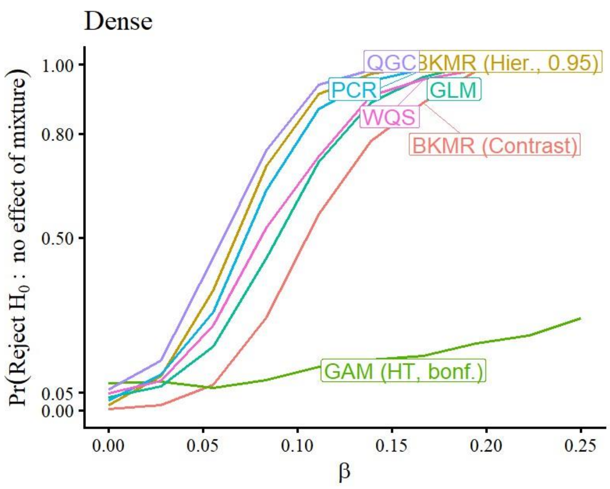

Figure S18: For each method (curve), the y-value is the probability of rejecting H_0: the overall mixture is not associated with the response, for different strengths of association β (x-axis). When $\beta = 0$, the value of the curve is the estimated Type I error probability. The targeted Type I error rate was 0.05. When $\beta \neq 0$, the value of the curve is the power of the hypothesis test. Better methods have Type I error rate at most 0.05, and power curve above other methods. The exposure-response function used is dense (all exposures affecting the response) with $p = 10$ exposures.

S2.3 Prediction error plots

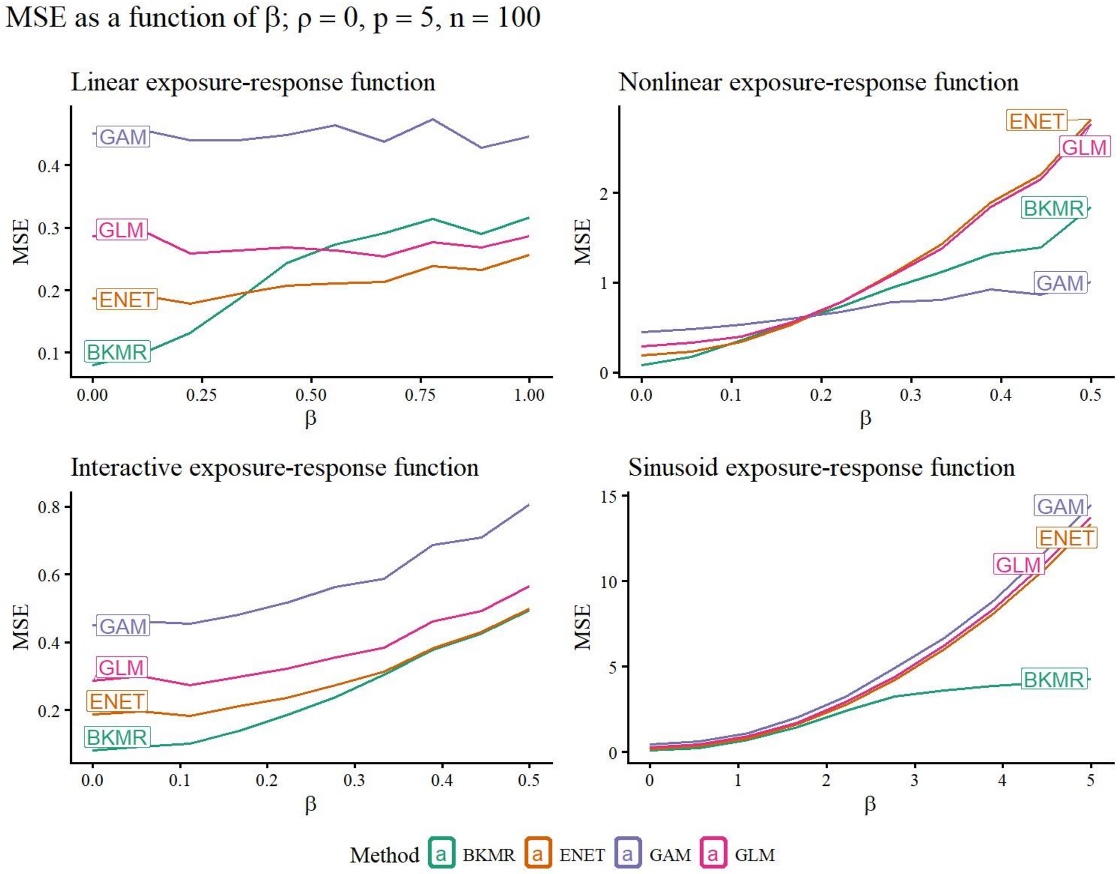

Figure S19: For each method (curve), the y-value is the prediction mean squared error (MSE) on new data, for different strengths of association β (x-axis). Better methods have lower MSE curves. The new data are generated from the same exposure-response functions as the data used to fit the model, but have no random variation added. Exposure-response functions used are linear, nonlinear, linear interaction, and sinusoid (nonlinear interaction).

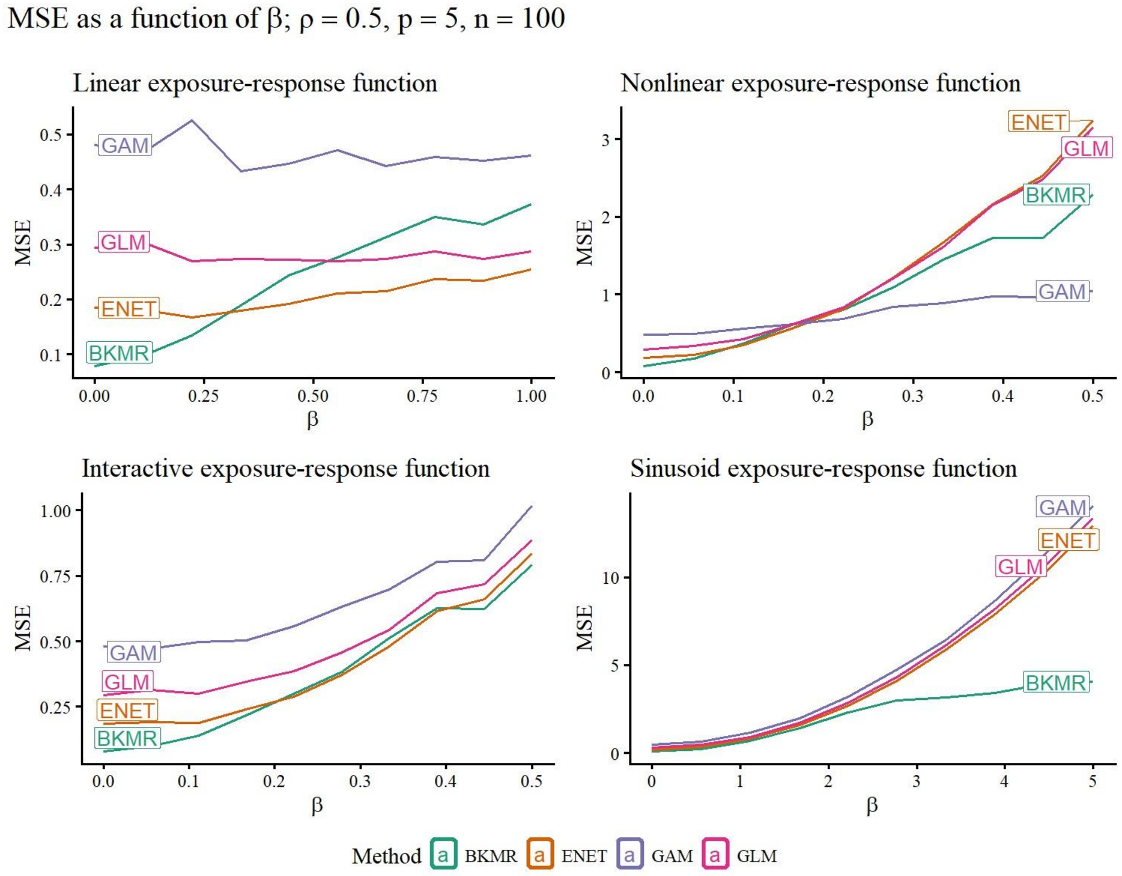

Figure S20: For each method (curve), the y-value is the prediction mean squared error (MSE) on new data, for different strengths of association β (x-axis). Better methods have lower MSE curves. The new data are generated from the same exposure-response functions as the data used to fit the model, but have no random variation added. Exposure-response functions used are linear, nonlinear, linear interaction, and sinusoid (nonlinear interaction).

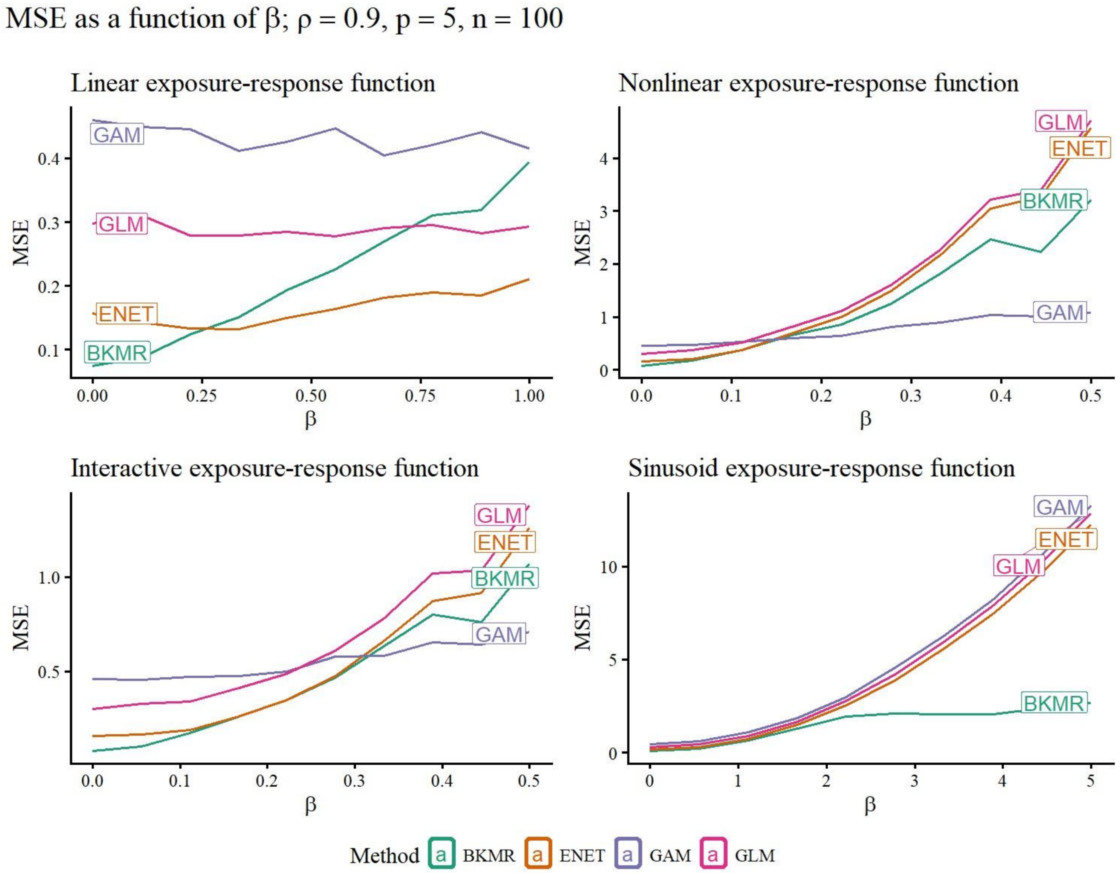

Figure S21: For each method (curve), the y-value is the prediction mean squared error (MSE) on new data, for different strengths of association β (x-axis). Better methods have lower MSE curves. The new data are generated from the same exposure-response functions as the data used to fit the model, but have no random variation added. Exposure-response functions used are linear, nonlinear, linear interaction, and sinusoid (nonlinear interaction).

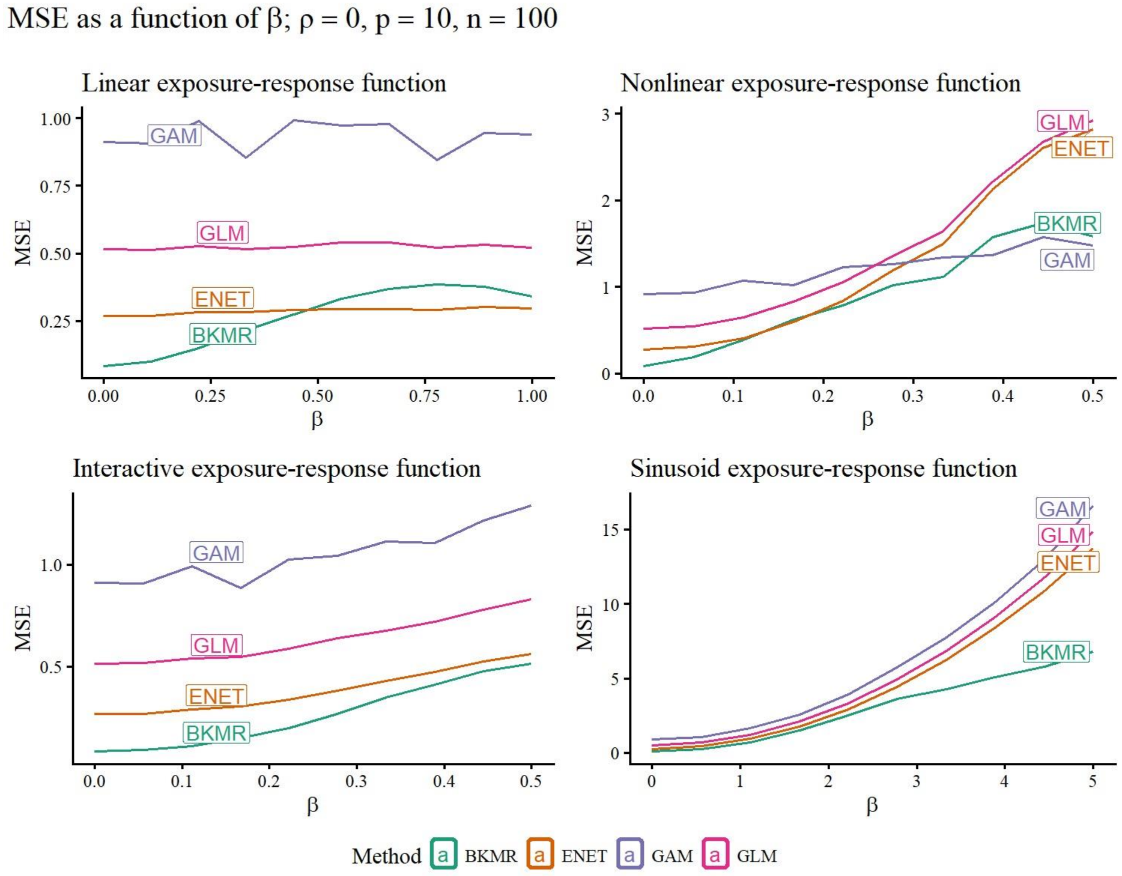

Figure S22: For each method (curve), the y-value is the prediction mean squared error (MSE) on new data, for different strengths of association β (x-axis). Better methods have lower MSE curves. The new data are generated from the same exposure-response functions as the data used to fit the model, but have no random variation added. Exposure-response functions used are linear, nonlinear, linear interaction, and sinusoid (nonlinear interaction).

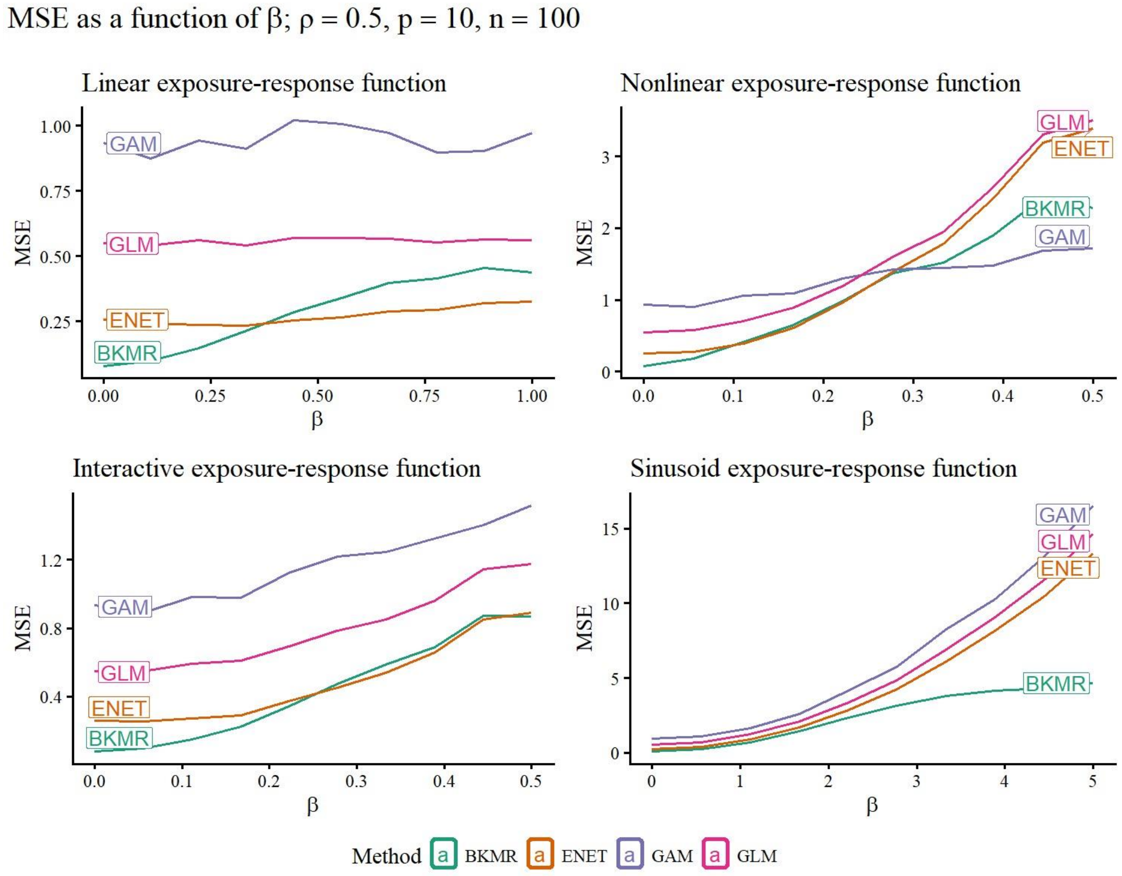

Figure S23: For each method (curve), the y-value is the prediction mean squared error (MSE) on new data, for different strengths of association β (x-axis). Better methods have lower MSE curves. The new data are generated from the same exposure-response functions as the data used to fit the model, but have no random variation added. Exposure-response functions used are linear, nonlinear, linear interaction, and sinusoid (nonlinear interaction).

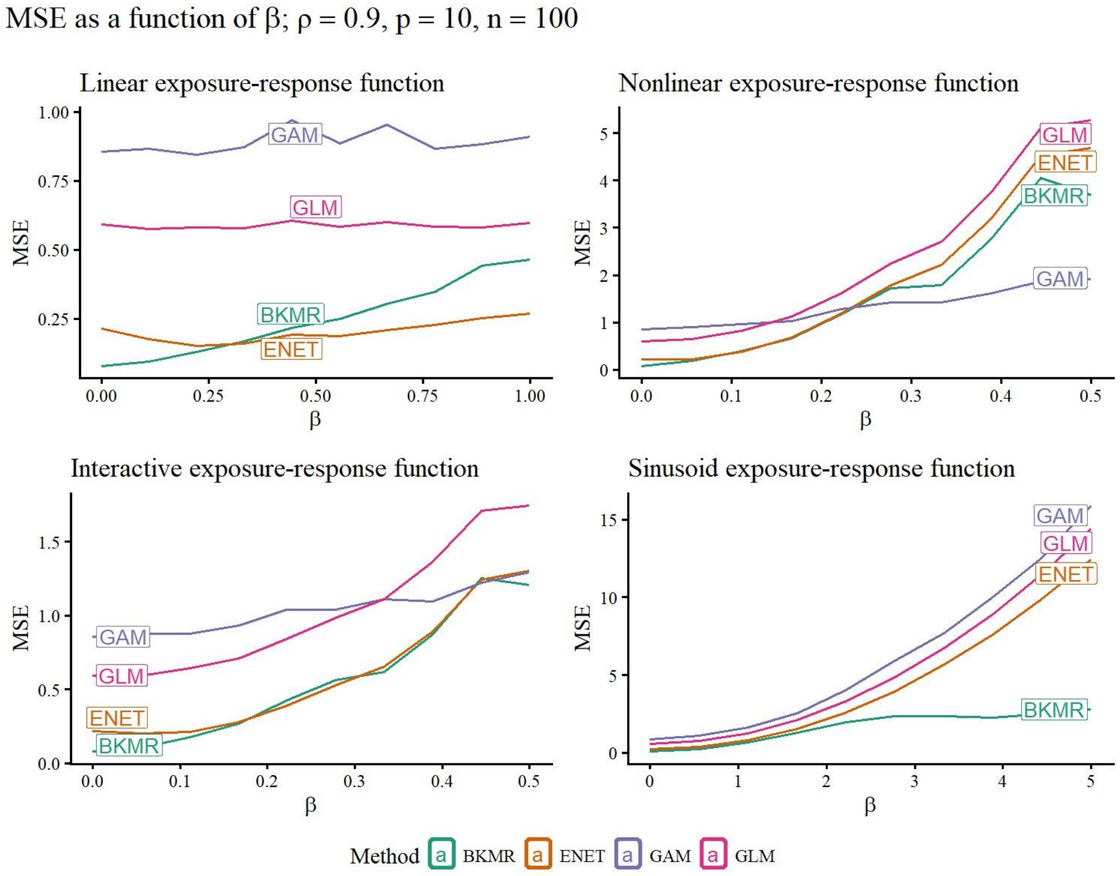

Figure S24: For each method (curve), the y-value is the prediction mean squared error (MSE) on new data, for different strengths of association β (x-axis). Better methods have lower MSE curves. The new data are generated from the same exposure-response functions as the data used to fit the model, but have no random variation added. Exposure-response functions used are linear, nonlinear, linear interaction, and sinusoid (nonlinear interaction).

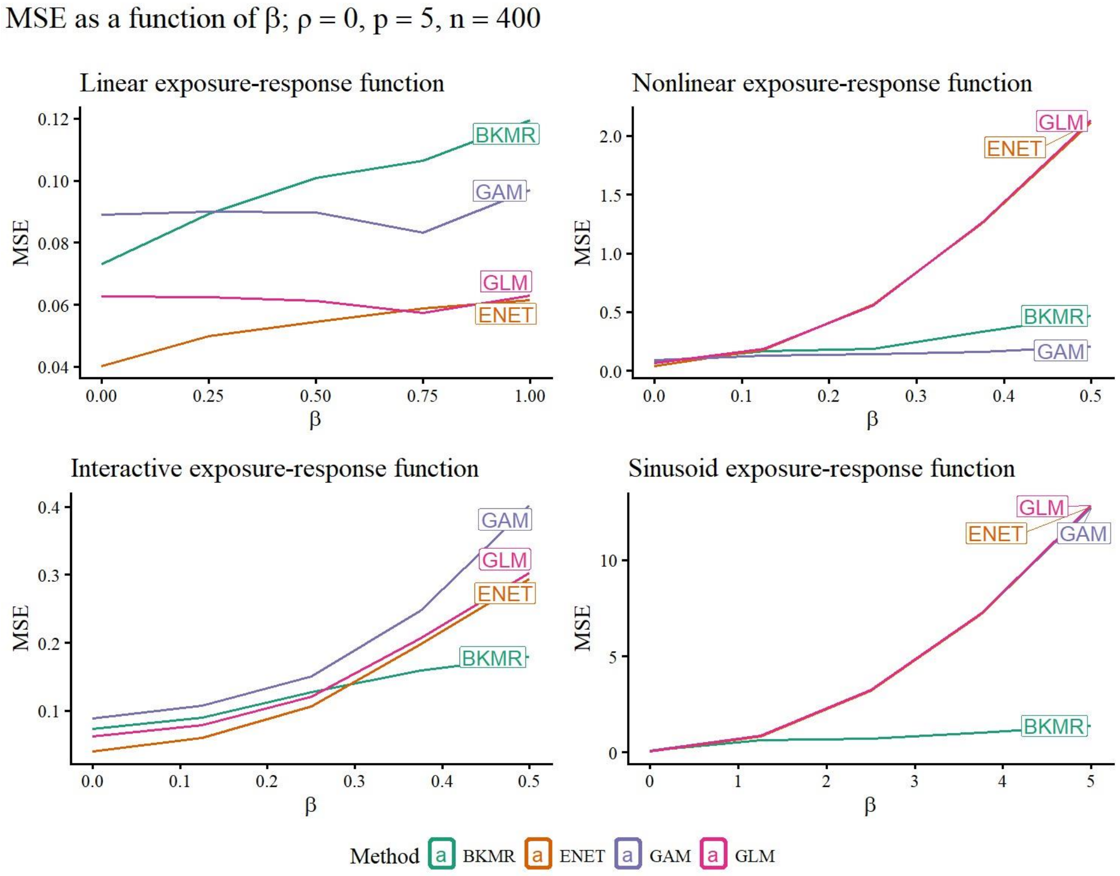

Figure S25: For each method (curve), the y-value is the prediction mean squared error (MSE) on new data, for different strengths of association β (x-axis). Better methods have lower MSE curves. The new data are generated from the same exposure-response functions as the data used to fit the model, but have no random variation added. Exposure-response functions used are linear, nonlinear, linear interaction, and sinusoid (nonlinear interaction).

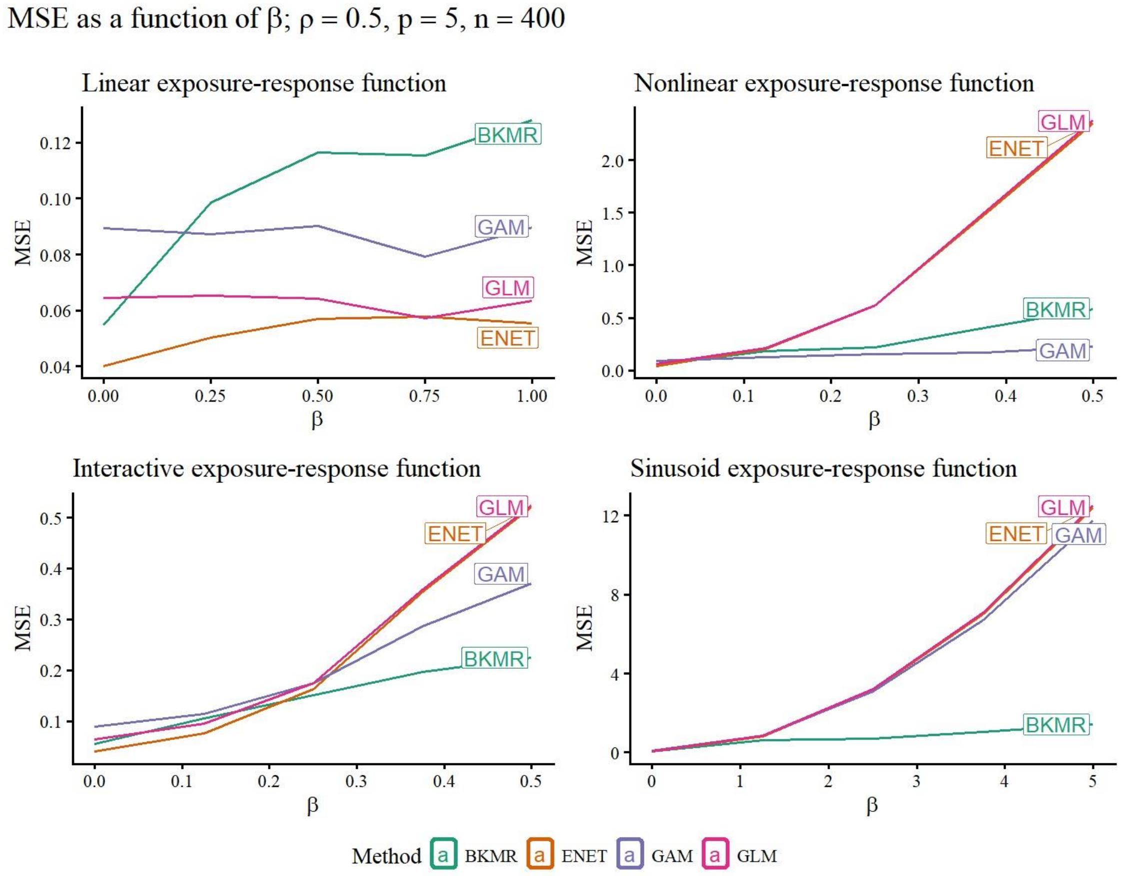

Figure S26: For each method (curve), the y-value is the prediction mean squared error (MSE) on new data, for different strengths of association β (x-axis). Better methods have lower MSE curves. The new data are generated from the same exposure-response functions as the data used to fit the model, but have no random variation added. Exposure-response functions used are linear, nonlinear, linear interaction, and sinusoid (nonlinear interaction).

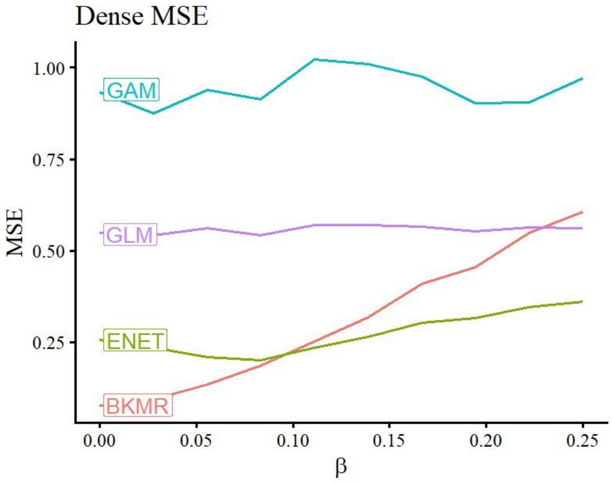

Figure S27: For each method (curve), the y-value is the prediction mean squared error (MSE) on new data, for different strengths of association β (x-axis). Better methods have lower MSE curves. The new data are generated from the same exposure-response functions as the data used to fit the model, but have no random variation added. The exposure-response function used is dense (all exposures affecting the response) with p = 10 exposures.

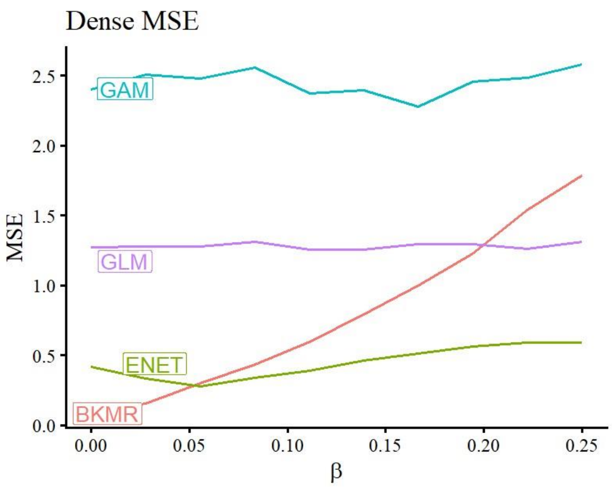

Figure S28: For each method (curve), the y-value is the prediction mean squared error (MSE) on new data, for different strengths of association β (x-axis). Better methods have lower MSE curves. The new data are generated from the same exposure-response functions as the data used to fit the model, but have no random variation added. The exposure-response function used is dense (all exposures affecting the response) with $p = 20$ exposures.

S3 Results Tables

This section provides results tables for all scenarios. The results tables display results for the value of β which maximized the standard deviation of probability of rejecting H_0 in that scenario, among methods displayed in the table. BKMR with hierarchical variable selection's results are not displayed as the group PIP was essentially always greater than 0.5 in all scenarios tried. The null distribution of the group PIP for different values of p, with default tuning, is displayed in Figure S29.

S3.1 Individual component hypothesis tests results tables

	β	GLM	GAM (HT)	GAM (VS)	ENET	BKMR (0.50)	BKMR (0.95)
Lin.	0.33	0.36	0.37	0.82	0.91	0.68	0.07
N.L	0.06	0.23	0.24	0.66	0.76	0.50	0.03
Int.	0.33	0.34	0.34	0.75	0.88	0.66	0.09
Sine	5.00	0.07	0.13	0.61	0.23	0.96	0.92
None	0.00	0.03	0.05	0.43	0.41	0.28	0.00

Table S1: Individual component hypothesis test performance for $n = 100$, $p = 5$, $\rho = 0$. Each row corresponds to a true exposure-response relationship (linear, nonlinear, linear interaction, sinusoidal, or none) along with a parameter β that controls the strength of the exposure-response relationship, where $\beta = 0$ corresponds to no relationship, and higher values correspond to stronger relationships. The columns for each method display the probability of rejecting the null hypothesis H_0: no association between treatment variable a_1 and the expected response y, estimated using Monte Carlo simulation. The targeted Type I error rate was 0.05.

	β	GLM	GAM (HT)	GAM (VS)	ENET	BKMR (0.50)	BKMR (0.95)
Lin.	0.44	0.42	0.44	0.85	0.92	0.81	0.14
N.L	0.11	0.38	0.40	0.76	0.84	0.69	0.17
Int.	0.22	0.26	0.30	0.72	0.83	0.65	0.09
Sine	5.00	0.09	0.28	0.70	0.16	0.99	0.95
None	0.00	0.05	0.07	0.41	0.38	0.30	0.00

Table S2: Individual component hypothesis test performance for $n = 100$, $p = 5$, $\rho = 0.5$. Each row corresponds to a true exposure-response relationship (linear, nonlinear, linear interaction, sinusoidal, or none) along with a parameter β that controls the strength of the exposure-response relationship, where $\beta = 0$ corresponds to no relationship, and higher values correspond to stronger relationships. The columns for each method display the probability of rejecting the null hypothesis H_0: no association between treatment variable a_1 and the expected response y, estimated using Monte Carlo simulation. The targeted Type I error rate was 0.05.

	β	GLM	GAM (HT)	GAM (VS)	ENET	BKMR (0.50)	BKMR (0.95)
Lin.	0.67	0.20	0.21	0.71	0.88	0.70	0.05
N.L	0.22	0.35	0.39	0.83	0.90	0.80	0.17
Int.	0.33	0.19	0.24	0.74	0.83	0.73	0.06
Sine	3.33	0.05	0.19	0.57	0.18	0.98	0.86
None	0.00	0.06	0.07	0.42	0.33	0.36	0.00

Table S3: Individual component hypothesis test performance for $n = 100$, $p = 5$, $\rho = 0.9$. Each row corresponds to a true exposure-response relationship (linear, nonlinear, linear interaction, sinusoidal, or none) along with a parameter β that controls the strength of the exposure-response relationship, where $\beta = 0$ corresponds to no relationship, and higher values correspond to stronger relationships. The columns for each method display the probability of rejecting the null hypothesis H_0: no association between treatment variable a_1 and the expected response y, estimated using Monte Carlo simulation. The targeted Type I error rate was 0.05.

	β	GLM	GAM (HT)	GAM (VS)	ENET	BKMR (0.50)	BKMR (0.95)
Lin.	0.44	0.57	0.58	0.91	0.96	0.76	0.12
N.L	0.11	0.49	0.52	0.86	0.91	0.67	0.12
Int.	0.33	0.31	0.34	0.75	0.86	0.56	0.05
Sine	4.44	0.04	0.13	0.57	0.17	0.80	0.60
None	0.00	0.06	0.09	0.42	0.36	0.25	0.00

Table S4: Individual component hypothesis test performance for $n = 100$, $p = 10$, $\rho = 0$. Each row corresponds to a true exposure-response relationship (linear, nonlinear, linear interaction, sinusoidal, or none) along with a parameter β that controls the strength of the exposure-response relationship, where $\beta = 0$ corresponds to no relationship, and higher values correspond to stronger relationships. The columns for each method display the probability of rejecting the null hypothesis H_0: no association between treatment variable a_1 and the expected response y, estimated using Monte Carlo simulation. The targeted Type I error rate was 0.05.

	β	GLM	GAM (HT)	GAM (VS)	ENET	BKMR (0.50)	BKMR (0.95)
Lin.	0.44	0.35	0.39	0.80	0.88	0.68	0.08
N.L	0.11	0.30	0.34	0.74	0.84	0.56	0.06
Int.	0.33	0.34	0.32	0.76	0.89	0.64	0.09
Sine	5.00	0.07	0.26	0.62	0.15	0.96	0.88
None	0.00	0.04	0.06	0.40	0.32	0.25	0.00

Table S5: Individual component hypothesis test performance for $n = 100$, $p = 10$, $\rho = 0.5$. Each row corresponds to a true exposure-response relationship (linear, nonlinear, linear interaction, sinusoidal, or none) along with a parameter β that controls the strength of the exposure-response relationship, where $\beta = 0$ corresponds to no relationship, and higher values correspond to stronger relationships. The columns for each method display the probability of rejecting the null hypothesis H_0: no association between treatment variable a_1 and the expected response y, estimated using Monte Carlo simulation. The targeted Type I error rate was 0.05.

	β	GLM	GAM (HT)	GAM (VS)	ENET	BKMR (0.50)	BKMR (0.95)
Lin.	0.89	0.27	0.27	0.77	0.93	0.55	0.03
N.L	0.28	0.37	0.41	0.82	0.90	0.66	0.12
Int.	0.44	0.25	0.26	0.69	0.82	0.55	0.04
Sine	5.00	0.06	0.20	0.56	0.12	1.00	1.00
None	0.00	0.04	0.05	0.40	0.24	0.22	0.00

Table S6: Individual component hypothesis test performance for $n = 100$, $p = 10$, $\rho = 0.9$.
Each row corresponds to a true exposure-response relationship (linear, nonlinear, linear interaction, sinusoidal, or none) along with a parameter β that controls the strength of the exposure-response relationship, where $\beta = 0$ corresponds to no relationship, and higher values correspond to stronger relationships. The columns for each method display the probability of rejecting the null hypothesis H_0: no association between treatment variable a_1 and the expected response y, estimated using Monte Carlo simulation. The targeted Type I error rate was 0.05.

	β	GLM	GAM (HT)	GAM (VS)	ENET	BKMR (0.50)	BKMR (0.95)
Lin.	0.25	0.69	0.69	0.93	0.96	0.82	0.22
N.L	0.12	0.96	0.98	1.00	1.00	0.98	0.87
Int.	0.12	0.22	0.21	0.69	0.79	0.50	0.04
Sine	5.00	0.13	0.43	0.81	0.06	1.00	1.00
None	0.00	0.06	0.06	0.48	0.34	0.30	0.00

Table S7: Individual component hypothesis test performance for $n = 400$, $p = 5$, $\rho = 0$. Each row corresponds to a true exposure-response relationship (linear, nonlinear, linear interaction, sinusoidal, or none) along with a parameter β that controls the strength of the exposure-response relationship, where $\beta = 0$ corresponds to no relationship, and higher values correspond to stronger relationships. The columns for each method display the probability of rejecting the null hypothesis H_0: no association between treatment variable a_1 and the expected response y, estimated using Monte Carlo simulation. The targeted Type I error rate was 0.05.

	β	GLM	GAM (HT)	GAM (VS)	ENET	BKMR (0.50)	BKMR (0.95)
Lin.	0.25	0.53	0.54	0.88	0.95	0.80	0.14
N.L	0.12	0.94	0.95	0.99	1.00	0.98	0.81
Int.	0.12	0.34	0.34	0.82	0.89	0.69	0.09
Sine	5.00	0.18	0.61	0.92	0.03	1.00	1.00
None	0.00	0.06	0.07	0.43	0.36	0.42	0.00

Table S8: Individual component hypothesis test performance for $n = 400$, $p = 4$, $\rho = 0.5$. Each row corresponds to a true exposure-response relationship (linear, nonlinear, linear interaction, sinusoidal, or none) along with a parameter β that controls the strength of the exposure-response relationship, where $\beta = 0$ corresponds to no relationship, and higher values correspond to stronger relationships. The columns for each method display the probability of rejecting the null hypothesis H_0: no association between treatment variable a_1 and the expected response y, estimated using Monte Carlo simulation. The targeted Type I error rate was 0.05.

S3.2 Whole-mixture hypothesis test results tables

	β	GLM	GAM (HT, bonf.)	PCR	BKMR (Contrast)	BKMR (Hier., 0.95)	WQS (Pos.)	QGC
Lin.	0.56	0.82	0.76	0.79	0.28	0.70	0.47	0.47
N.L.	0.17	0.88	0.85	0.84	0.16	0.84	0.35	0.34
Int.	0.50	0.69	0.58	0.66	0.18	0.56	0.39	0.41
Sine	5.00	0.09	0.14	0.09	0.42	0.14	0.09	0.14
None	0.00	0.06	0.06	0.07	0.00	0.04	0.05	0.06

Table S9: Whole-mixture performance for $n = 100$, $p = 5$, $\rho = 0$. Each row corresponds to a true exposure-response relationship (linear, nonlinear, linear interaction, sinusoidal, or none) along with a parameter β that controls the strength of the exposure-response relationship, where $\beta = 0$ corresponds to no relationship, and higher values correspond to stronger relationships. The columns for each method display the probability of rejecting the null hypothesis H_0: no association between any treatment variables $a_1,\ldots,a_p$ and the expected response y, estimated using Monte Carlo simulation. The targeted Type I error rate was 0.05.

	β	GLM	GAM (HT, bonf.)	PCR	BKMR (Contrast)	BKMR (Hier., 0.95)	WQS (Pos.)	QGC
Lin.	0.44	0.80	0.37	0.84	0.41	0.84	0.59	0.79
N.L.	0.17	0.94	0.70	0.95	0.35	0.96	0.70	0.84
Int.	0.39	0.87	0.55	0.92	0.29	0.90	0.56	0.79
Sine	5.00	0.14	0.24	0.16	0.56	0.34	0.22	0.43
None	0.00	0.05	0.09	0.04	0.00	0.03	0.04	0.04

Table S10: Whole-mixture performance for $n = 100$, $p = 5$, $\rho = 0.5$. Each row corresponds to a true exposure-response relationship (linear, nonlinear, linear interaction, sinusoidal, or none) along with a parameter β that controls the strength of the exposure-response relationship, where $\beta = 0$ corresponds to no relationship, and higher values correspond to stronger relationships. The columns for each method display the probability of rejecting the null hypothesis H_0: no association between any treatment variables $a_1,\ldots,a_p$ and the expected response y, estimated using Monte Carlo simulation. The targeted Type I error rate was 0.05.

	β	GLM	GAM (HT, bonf.)	PCR	BKMR (Contrast)	BKMR (Hier., 0.95)	WQS (Pos.)	QGC
Lin.	0.44	0.89	0.14	0.97	0.61	0.97	0.78	0.92
N.L.	0.11	0.74	0.20	0.91	0.20	0.88	0.48	0.67
Int.	0.28	0.86	0.22	0.96	0.26	0.94	0.58	0.81
Sine	5.00	0.26	0.31	0.35	0.70	0.85	0.43	0.78
None	0.00	0.03	0.08	0.05	0.00	0.03	0.03	0.04

Table S11: Whole-mixture performance for $n = 100, p = 5, \rho = 0.9$. Each row corresponds to a true exposure-response relationship (linear, nonlinear, linear interaction, sinusoidal, or none) along with a parameter β that controls the strength of the exposure-response relationship, where $\beta = 0$ corresponds to no relationship, and higher values correspond to stronger relationships. The columns for each method display the probability of rejecting the null hypothesis H_0: no association between any treatment variables $a_1,\ldots,a_p$ and the expected response y, estimated using Monte Carlo simulation. The targeted Type I error rate was 0.05.

	β	GLM	GAM (HT, bonf.)	PCR	BKMR (Contrast)	BKMR (Hier., 0.95)	WQS (Pos.)	QGC
Lin.	0.67	0.85	0.86	0.81	0.24	0.80	0.52	0.37
N.L.	0.17	0.80	0.80	0.76	0.08	0.76	0.27	0.20
Int.	0.50	0.56	0.55	0.52	0.06	0.48	0.32	0.22
Sine	5.00	0.05	0.13	0.06	0.24	0.10	0.08	0.11
None	0.00	0.03	0.06	0.04	0.00	0.02	0.07	0.04

Table S12: Whole-mixture performance for $n = 100$, $p = 10$, $\rho = 0$. Each row corresponds to a true exposure-response relationship (linear, nonlinear, linear interaction, sinusoidal, or none) along with a parameter β that controls the strength of the exposure-response relationship, where $\beta = 0$ corresponds to no relationship, and higher values correspond to stronger relationships. The columns for each method display the probability of rejecting the null hypothesis H_0: no association between any treatment variables $a_1,\ldots,a_p$ and the expected response y, estimated using Monte Carlo simulation. The targeted Type I error rate was 0.05.

	β	GLM	GAM (HT, bonf.)	PCR	BKMR (Contrast)	BKMR (Hier., 0.95)	WQS (Pos.)	QGC
Lin.	0.44	0.70	0.26	0.76	0.37	0.83	0.62	0.80
N.L.	0.17	0.87	0.54	0.90	0.30	0.93	0.64	0.83
Int.	0.39	0.82	0.38	0.86	0.23	0.89	0.62	0.80
Sine	5.00	0.10	0.20	0.12	0.44	0.30	0.22	0.42
Opp.	-0.78	0.88	0.81	0.68	0.04	0.58	0.22	0.14
None	0.00	0.04	0.08	0.03	0.00	0.01	0.05	0.06

Table S13: Whole-mixture performance for $n = 100$, $p = 10$, $\rho = 0.5$. Each row corresponds to a true exposure-response relationship (linear, nonlinear, linear interaction, sinusoidal, opposite, or none) along with a parameter β that controls the strength of the exposure-response relationship, where $\beta = 0$ corresponds to no relationship, and higher values correspond to stronger relationships, except for the opposite-effects scenario. In the opposite effects scenario, $E(y|a_1,a_2) = \beta a_1 + a_2$. The columns for each method display the probability of rejecting the null hypothesis H_0: no association between any treatment variables $a_1,\ldots,a_p$ and the expected response y, estimated using Monte Carlo simulation. The targeted Type I error rate was 0.05.

	β	GLM	GAM (HT, bonf.)	PCR	BKMR (Contrast)	BKMR (Hier., 0.95)	WQS (Pos.)	QGC
Lin.	0.67	0.98	0.09	1.00	0.92	1.00	0.96	1.00
N.L.	0.17	0.91	0.16	0.99	0.35	0.99	0.77	0.92
Int.	0.33	0.90	0.14	0.98	0.29	0.97	0.72	0.86
Sine	5.00	0.17	0.25	0.38	0.67	0.82	0.49	0.74
None	0.00	0.04	0.05	0.05	0.01	0.04	0.06	0.05

Table S14: Whole-mixture performance for $n = 100$, $p = 10$, $\rho = 0.9$. Each row corresponds to a true exposure-response relationship (linear, nonlinear, linear interaction, sinusoidal, or none) along with a parameter β that controls the strength of the exposure-response relationship, where $\beta = 0$ corresponds to no relationship, and higher values correspond to stronger relationships. The columns for each method display the probability of rejecting the null hypothesis H_0: no association between any treatment variables $a_1,\ldots,a_p$ and the expected response y, estimated using Monte Carlo simulation. The targeted Type I error rate was 0.05.

	β	GLM	GAM (HT, bonf.)	PCR	BKMR (Contrast)	BKMR (Hier., 0.95)	WQS (Pos.)	QGC
Lin.	0.25	0.76	0.68	0.74	0.20	0.56	0.48	0.44
N.L.	0.12	1.00	1.00	0.99	0.48	1.00	0.76	0.66
Int.	0.25	0.76	0.64	0.70	0.16	0.52	0.41	0.47
Sine	3.75	0.10	0.31	0.09	0.95	0.99	0.11	0.36
None	0.00	0.04	0.06	0.06	0.00	0.02	0.04	0.06

Table S15: Whole-mixture performance for $n = 400$, $p = 5$, $\rho = 0$. Each row corresponds to a true exposure-response relationship (linear, nonlinear, linear interaction, sinusoidal, or none) along with a parameter β that controls the strength of the exposure-response relationship, where $\beta = 0$ corresponds to no relationship, and higher values correspond to stronger relationships. The columns for each method display the probability of rejecting the null hypothesis H_0: no association between any treatment variables $a_1,\ldots,a_p$ and the expected response y, estimated using Monte Carlo simulation. The targeted Type I error rate was 0.05.

	p	β	GLM	GAM (HT, bonf.)	PCR	BKMR (Contrast)	BKMR (Hier., 0.95)	WQS (Pos.)	QGC
Lin.	10	0.08	0.44	0.09	0.64	0.26	0.70	0.53	0.75
Lin.	20	0.06	0.49	0.14	0.77	0.45	0.89	0.71	0.89
None	10	0.00	0.04	0.08	0.03	0.00	0.01	0.05	0.06
None	20	0.00	0.08	0.14	0.07	0.00	0.04	0.08	0.08

Table S17: Whole-mixture performance for the dense scenarios with $n = 100$, $\rho = 0.5$. Each row corresponds to a true exposure-response relationship (linear or none) along with a parameter β that controls the strength of the exposure-response relationship, where $\beta = 0$ corresponds to no relationship, and higher values correspond to stronger relationships. The columns for each method display the probability of rejecting the null hypothesis H_0: no association between any treatment variables $a_1,\ldots,a_p$ and the expected response y, estimated using Monte Carlo simulation. The targeted Type I error rate was 0.05.

	β	GLM	GAM (HT, bonf.)	PCR	BKMR (Contrast)	BKMR (Hier., 0.95)	WQS (Pos.)	QGC
Lin.	0.25	0.90	0.44	0.89	0.50	0.89	0.75	0.88
N.L.	0.12	1.00	0.98	1.00	0.58	1.00	0.92	1.00
Int.	0.12	0.66	0.28	0.68	0.10	0.62	0.38	0.52
Sine	3.75	0.34	0.68	0.41	0.96	0.99	0.48	0.93
None	0.00	0.06	0.06	0.06	0.00	0.04	0.10	0.06

Table S16: Whole-mixture performance for $n = 400, p = 5, \rho = 0.5$. Each row corresponds to a true exposure-response relationship (linear, nonlinear, linear interaction, sinusoidal, or none) along with a parameter β that controls the strength of the exposure-response relationship, where $\beta = 0$ corresponds to no relationship, and higher values correspond to stronger relationships. The columns for each method display the probability of rejecting the null hypothesis H_0: no association between any treatment variables $a_1,\ldots,a_p$ and the expected response y, estimated using Monte Carlo simulation. The targeted Type I error rate was 0.05.

S4 Other supporting information

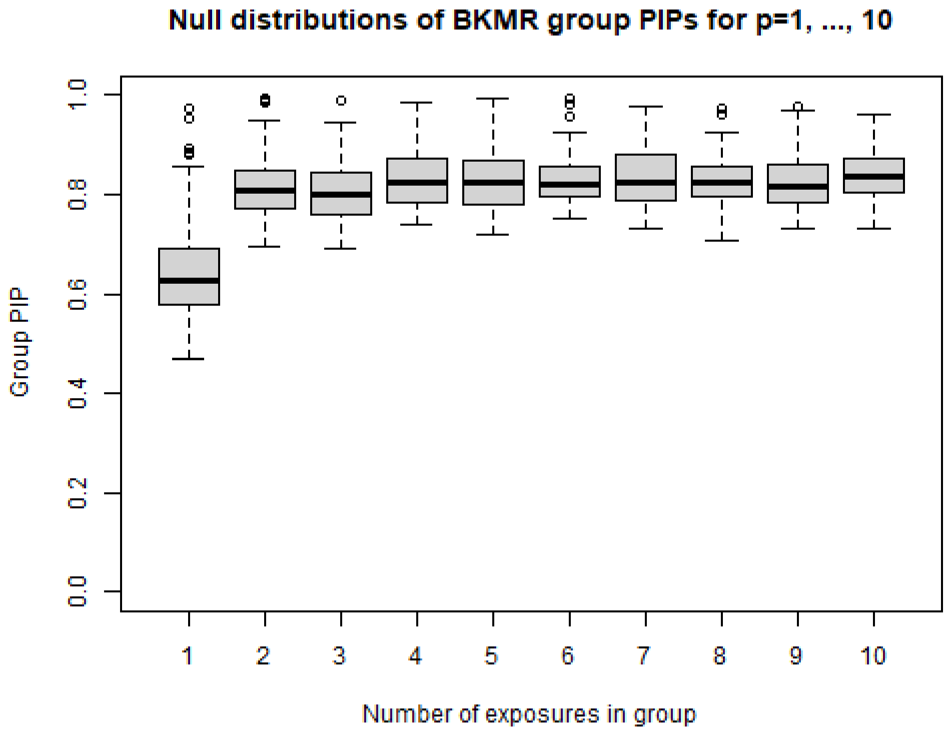

Figure S29: Null distribution of BKMR hierarchical variable selection group PIPs using package-default settings, with $n = 100$, $\rho = 0.5$, 100 simulated data sets, and 20,000 MCMC iterations with 10,000 discarded as burn-in. The number of exposure variables in the simulated data sets p ranged from 1 to 10, all of which were included in a single group.

References

[1] S. N. Wood. “Fast stable restricted maximum likelihood and marginal likelihood estimation of semiparametric generalized linear models”. In: *Journal of the Royal Statistical Society (B)* 73.1 (2011), pp. 3–36.

[2] Simon N Wood. *Generalized additive models: an introduction with R*. CRC press, 2017.

[3] Jennifer F Bobb, Linda Valeri, Birgit Claus Henn, et al. “Bayesian kernel machine regression for estimating the health effects of multi-pollutant mixtures”. In: *Biostatistics* 16.3 (2015), pp. 493–508.

[4] Jennifer F. Bobb. *bkmr: Bayesian Kernel Machine Regression*. R package version 0.2.2. 2022. URL: https://CRAN.R-project.org/package=bkmr.

[5] Nina Lazarevic, Luke D Knibbs, Peter D Sly, and Adrian G Barnett. “Performance of variable and function selection methods for estimating the nonlinear health effects of correlated chemical mixtures: A simulation study”. In: *Statistics in Medicine* 39.27 (2020), pp. 3947–3967.

[6] Jerome Friedman, Robert Tibshirani, and Trevor Hastie. “Regularization Paths for Generalized Linear Models via Coordinate Descent”. In: *Journal of Statistical Software* 33.1 (2010), pp. 1–22. DOI: 10.18637/jss.v033.i01.

[7] Max Kuhn. “Building Predictive Models in R Using the caret Package”. In: *Journal of Statistical Software* 28.5 (2008). R package version 6.0.94, pp. 1–26. DOI: 10.18637/jss.v028.i05. URL: https://www.jstatsoft.org/index.php/jss/article/ view/v028i05.

[8] Jennifer F Bobb, Birgit Claus Henn, Linda Valeri, and Brent A Coull. “Statistical software for analyzing the health effects of multiple concurrent exposures via Bayesian kernel machine regression”. In: *Environmental Health* 17.1 (2018), pp. 1–10.

[9] Stefano Renzetti, Paul Curtin, Allan C Just, Ghalib Bello, and Chris Gennings. *gWQS: Generalized Weighted Quantile Sum Regression*. R package version 3.0.4. 2021. URL: https://CRAN.R-project.org/package=gWQS.

[10] Alexander Keil. *qgcomp: Quantile G-Computation*. R package version 2.10.1. 2022. URL: https://CRAN.R-project.org/package=qgcomp.
